# The Golgi-localized sphingosine-1-phosphate phosphatase is indispensable for *Leishmania major*

**DOI:** 10.1038/s41598-022-20249-w

**Published:** 2022-09-26

**Authors:** Brian Okundaye, Neha Biyani, Samrat Moitra, Kai Zhang

**Affiliations:** 1 Department of Biological Sciences, Texas Tech University, Lubbock, TX 79409 USA; 2 Present Address: The Institute of Environmental and Human Health, Texas Tech University, Lubbock, TX 79409 USA; 3 Present Address: Lantern Pharma Inc., 1920 McKinney Ave., Dallas, TX 75201 USA

**Keywords:** Biochemistry, Cell biology, Microbiology, Molecular biology

## Abstract

Sphingosine-1-phosphate phosphatase (SPP) catalyzes the dephosphorylation of sphingosine-1-phosphate (S1P) into sphingosine, the reverse reaction of sphingosine kinase. In mammals, S1P acts as a potent bioactive molecule regulating cell proliferation, migration, and immunity. In *Leishmania*, S1P production is crucial for the synthesis of ethanolamine and choline phospholipids, and cell survival under stress conditions. To better understand the roles of S1P, we characterized a SPP ortholog in *Leishmania major* which displays activity towards S1P but not structurally related lipids such as ceramide-1-phosphate or lysophosphatidic acid. While this enzyme is found in the endoplasmic reticulum in mammalian cells, *L. major* SPP is localized at the Golgi apparatus. Importantly, chromosomal *SPP* alleles cannot be deleted from *L. major* even with the addition of a complementing episome, suggesting that endogenously expressed *SPP* is essential. Finally, *SPP* overexpression in *L. major* leads to a slower growth rate and heightened sensitivity to brefeldin A and sodium orthovanadate. Together, these results suggest that the equilibrium between S1P and sphingosine is vital for the function of Golgi apparatus in *Leishmania*.

## Introduction

Trypanosomatid parasites of the genus *Leishmania* are the causative agents for leishmaniasis, which can manifest from self-limiting skin ulcers to disfiguring mucocutaneous lesions and life-threatening visceral infections^1^. Leishmaniasis is transmitted through the bite of sandflies in the tropical and subtropical regions, mostly afflicting the poor, and is classified as a neglected tropical disease^1^. *Leishmania* parasites alternate between extracellular flagellated promastigotes in sandflies and intracellular non-flagellated amastigotes in macrophages. There is no vaccine and drugs are plagued with high toxicity and low efficacy^2^. Given the lack of means to control leishmaniasis, there is a clear need to develop new therapies targeting those unique cellular pathways that allow *Leishmania* parasites to survive in humans and sandflies.

**Figure 1:**
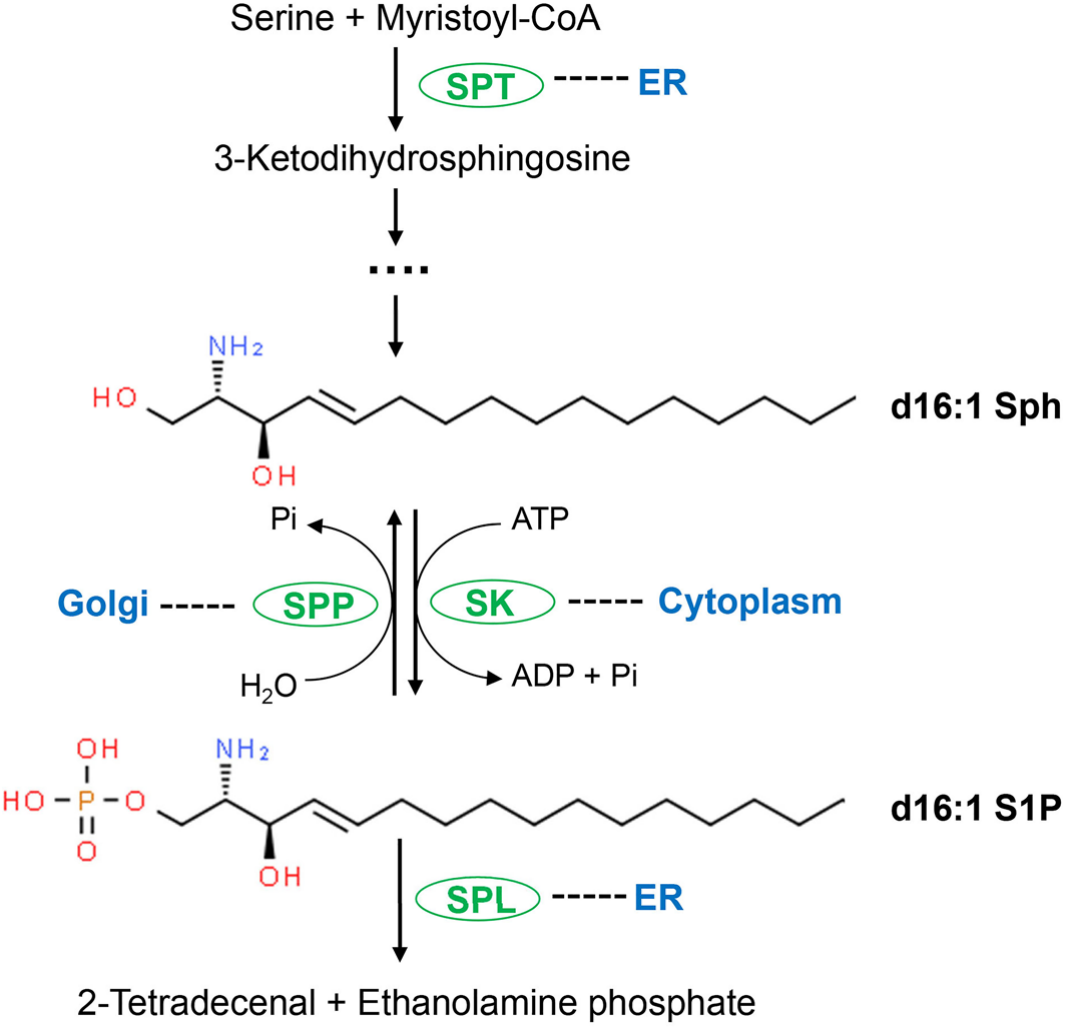
Metabolism of sphingosine (Sph) and sphingosine-1-phosphate (S1P) in *Leishmania major*. *SPT* serine palmitoyltransferase, *SK* sphingosine kinase, *SPP* sphingosine-1-phosphate phosphatase, *SPL* sphingosine-1-phosphate lyase. Cellular localizations of these enzymes in *Leishmania* are indicated.

Sphingolipids represent a diverse class of lipids ranging from basic building blocks in sphingoid bases or ceramide to complex glycosylated forms^3,4^. They are the structural components of lipid rafts, and their metabolism produces signaling molecules that modulate fundamental cellular processes^5,6^. In mammals, sphingosine-1-phosphate (S1P) interacts with G protein-coupled membrane receptors to control cell adhesion, migration, differentiation, and survival^7,8^. S1P also acts as an intracellular second messenger by regulating targets such as histone deacetylases, mitochondrial prohibitin 2, and tumor necrosis factor receptor-associated factor 2 while the dephosphorylation of S1P into sphingosine (Sph) promotes cell death or senescence^7,9^. Interconversions between S1P and Sph are catalyzed by sphingosine kinase (SK), which phosphorylates Sph into S1P^10,11^, and sphingosine-1-phosphate phosphatase (SPP)^12^ or other lipid phosphate phosphatase (LPP) that can hydrolyze S1P into Sph^13^ (Fig. 1). Through the action of sphingosine-1-phosphate lyase (SPL), S1P can also be irreversibly degraded into ethanolamine phosphate and fatty aldehyde, and contribute to the biosynthesis of phosphatidylethanolamine, in addition to its signaling functions (Fig. 1)^14–16^. Furthermore, S1P and Sph can be generated from the catabolism of sphingosylphosphorylcholine (by autotaxin)^17^ and ceramide (by ceramidases)^18^, respectively. The collective balance between S1P production and turnover constitutes a major determinant in mammalian cells’ response to heat shock and DNA damage, and the maturation of immune cells and vascular genesis^7,19,20^.

Functions of sphingolipids in *Leishmania* seem to differ substantially from those in mammals. Serine palmitoyltransferase (SPT) is the first enzyme of de novo sphingolipid biosynthesis responsible for condensing serine and a fatty acyl CoA (in *Leishmania*, mainly myristoyl-CoA) into 3-ketodihydrosphingosine^21^. *Leishmania major* mutants deficient for the catalytic subunit of SPT (*spt2*^−^) are viable and replicative despite the complete loss of sphingolipid synthesis^22,23^. They maintain normal detergent resistant membrane fractions but grow slower than wild type (WT) parasites and fail to differentiate into the infective metacyclic forms in stationary phase^22,23^. These phenotypes are similar to those of the *SPL* knockout mutant (*spl*^−^) which cannot break down S1P and both *spt2*^−^ and *spl*^−^ mutants can be fully rescued by the addition of ethanolamine to culture media, indicating that a major role of sphingoid base metabolism in *Leishmania* is to provide ethanolamine phosphate for the synthesis of ethanolamine and choline phospholipids^16,24^ (Fig. 1).

To investigate if S1P possesses other functions in *Leishmania*, the sphingosine kinase a (*SKa*) gene in *L. major* was deleted to generate the *ska*^−^ mutant^25^. Although *SKa* is the only recognizable SK homolog, lysate from *ska*^−^ promastigotes retains the activity to phosphorylate Sph and dihydrosphingosine at 50–60% of WT-levels, suggesting that *L. major*, like animals and yeasts, possesses more than one functional SK^25^. The partial loss of SK activity causes accumulation of cytotoxic sphingoid bases in *ska*^−^ leading to cell death in late log phase, which can be rescued through the addition of myriocin, a SPT inhibitor which blocks sphingoid base synthesis, to the media^25^. *Ska*^−^ mutants also show increased sensitivity to heat, acidic pH, and oxidants, and severely attenuated virulence in mice. Interestingly, the virulence defects of *ska*^−^ can only be reversed by the chromosomal knock-in but not episomal overexpression of *SKa*, implying that proper expression of this gene from its endogenous locus is essential for *L. major*^25^. The mechanism by which S1P impacts stress response in *Leishmania* awaits further investigation.

SPP, the enzyme catalyzing the dephosphorylation of S1P into Sph (the reverse reaction of SK), has yet to be characterized in trypanosomatids. Homologs of SPP can be found in the genomes of *Leishmania* spp, *Crithidia* spp, *Blechomonas* spp, *Endotrypanum* spp, and *Trypanosoma cruzi*, but not *Trypanosoma brucei* (https://tritrypdb.org/tritrypdb/app). In other eukaryotes including *Saccharomyces cerevisiae*, *Arabidopsis thaliana*, *Homo sapiens* and *Mus musculus*, SPP has been studied in the context of their relationship to SK^26–32^. In these organisms, SPP is an endoplasmic reticulum (ER) localized protein. Knockouts of YSR2 and YSR3, the SPP isoforms in *S. cerevisiae*, result in accumulation of S1P and/or dihydrosphingosine-1-phosphate but little change in viability or proliferation^32^. Overexpression of YSR2 or YSR3 is associated with increased vulnerability to heat shock and a tendency towards arrested development at the G1 phase^32^. Similar results were reported in knockout studies of *SPP* orthologs in *A. thaliana*^31^. Mice deficient of *SPP1* or *SPP2* are also viable, with deletion of SPP1 resulting in stunted growth, malformation of the skin, and poor survivorship to adulthood in contrast to mice deficient of *SPP2* which appear normal but have fewer β-cells in their pancreas compared to wild type mice^33,34^. In these organisms, SPPs are functionally redundant with LPPs which possess dephosphorylating activity towards lysophosphatidic acid, phosphatidic acid, ceramide-1-phosphate, as well as S1P and dihydrosphingosine-1-phosphate^9,35,36^. This would at least in part explain why SPP is nonessential in these organisms.

In this study, we investigated the role of a single SPP in *L. major* through attempted knockout and overexpression experiments. Our results revealed that *L. major* SPP can dephosphorylate fluorescently labeled S1P but not ceramide-1-phosphate or lysophosphatidic acid and is localized to the Golgi apparatus. Importantly, chromosomal *SPP* null mutants cannot be generated even in the presence of a *SPP*-containing episome, suggesting that *SPP* needs to be expressed from its endogenous locus to fulfill its full functions. In agreement with this notion, *SPP* overexpression led to delayed growth and increased sensitivity to brefeldin A, which inhibits intracellular protein trafficking from the ER to the Golgi apparatus. Thus, like *SKa*, the expression of *SPP* needs to be carefully regulated to ensure *Leishmania* survival. Taken together, these observations suggest that equilibrium between SPP and SKa expression is crucial for *Leishmania*. Future work would focus on the molecular target of S1P in *Leishmania* and evaluating the potential of sphingoid base metabolism in developing novel therapeutics.

## Results

### Identification of a functional *L. major* SPP

**Figure 2:**
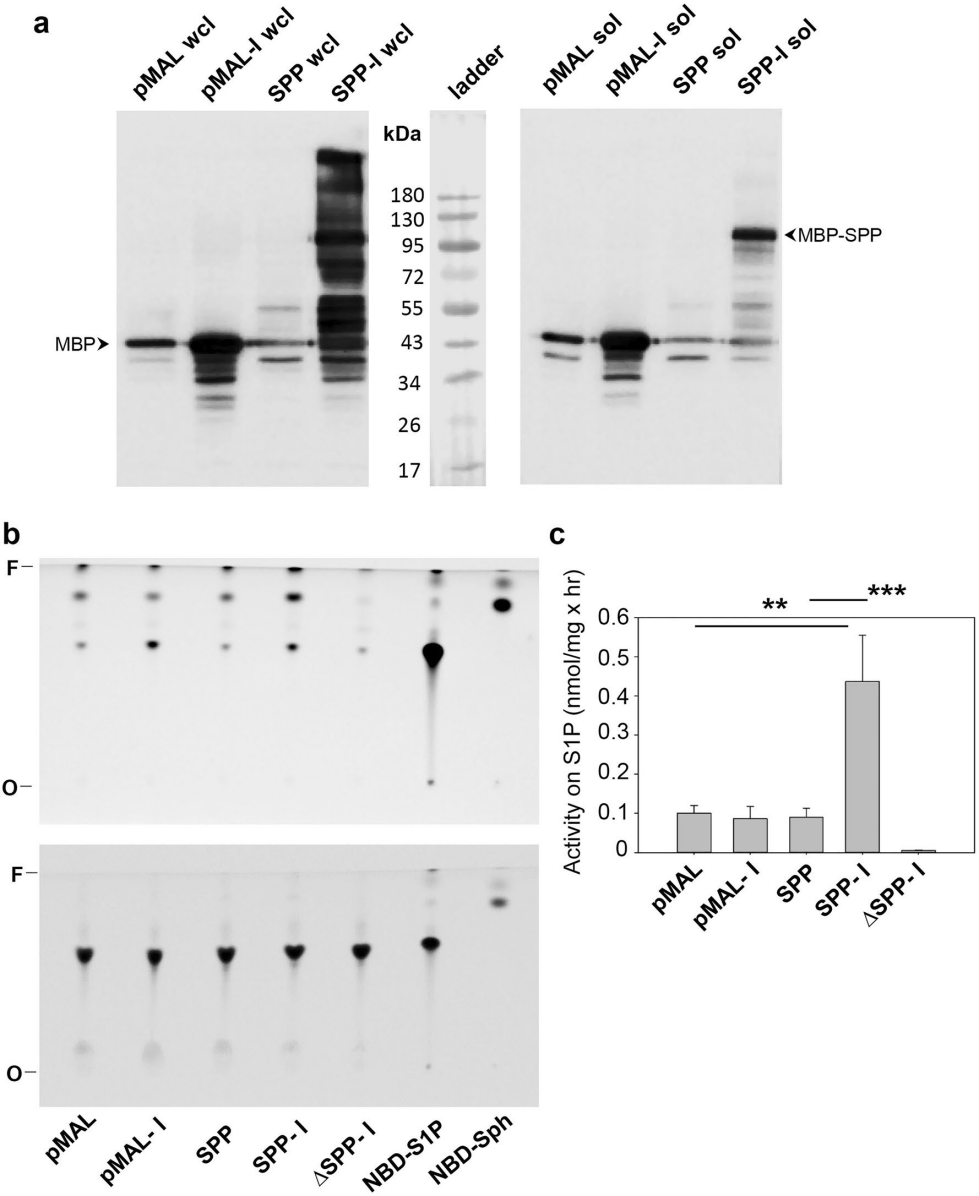
Recombinant *Leishmania major* SPP exhibits activity towards NBD-S1P. (**a**) *E. coli* lysates containing pMAL-MBP-SPP (SPP) or pMAL-c5x vector (pMAL) were subjected to western blot analysis using an anti-MBP antibody. Wcl: whole cell lysate; Sol: soluble lysate; I: induced with IPTG. Positions of MBP-SPP (112 kDa) and MBP (42.5 kDa) were indicated. Each lane contained 5 μg of protein (based on BCA assay). (**b**) Soluble lysates were incubated with NBD-S1P as indicated in “Methods”. After lipid extraction, the organic (top) and aqueous (bottom) fractions were analyzed by thin layer chromatography. Pure NBD-S1P and NBD-Sph were included as references. Boiled lysate of induced pMAL-SPP (SPP-I) was included as a control. *F* solvent front, *O* origin. (**c**) Quantitation of the enzymatic conversion of NBD-S1P into NBD-Sph by soluble *E. coli* lysates. Error bars represent standard deviations from three experiments. **p < 0.01. ***p < 0.001. The full-length blots for (**a**) are included in Supplemental Fig. S2.

A single *SPP* gene (Tritrydb gene ID: LmjF.32.2290; Genbank accession number: Q4Q546) was identified in the *L. major* genome with syntenic orthologs in several other trypanosomatids. The predicted *L. major* SPP protein (646 amino acids) is significantly larger than its orthologs in yeast, human, and mouse with N- and C-terminal extensions (Supplemental Fig. S1). It shows 24–27% identity to other SPPs and includes a putative active site motif “PST(S)H” at positions 296–299 (Supplemental Fig. S1). While many eukaryotes including *Saccharomyces cerevisiae*, *Homo sapiens*, *Mus musculus,* and *Arabidopsis thaliana* possess two SPP isoforms, only one putative SPP is identified in *L. major* and other trypanosomatids. To determine if *L. major* SPP possesses the expected enzymatic activity, we cloned the gene into a pMAL-c5x vector to generate a N-terminal maltose binding protein (MBP) tagged version of SPP (MBP-SPP), and the resulting pMAL-MBP-SPP plasmid was transformed into *Escherichia coli* BL21 cells. Western blot detected the presence of MBP-SPP (~ 112 kDa) in cell lysates after IPTG induction (Fig. 2a and Supplemental Fig. S2). Meanwhile, *E. coli* cells containing the empty pMAL-c5x vector produced free MBP (~ 42.5 kDa) (Fig. 2a and Supplemental Fig. S2).

When soluble lysates from *E. coli* BL21 cells were incubated with C18 NBD-S1P, a fluorescent analog of S1P, we detected a fluorescent lipid product in the organic fraction that migrated similarly as NBD-Sph on thin layer chromatography (TLC) (Fig. 2b). This process is likely enzymatic as boiled lysates had no activity. In comparison to those from uninduced cells and cells with empty pMAL-c5x vector, the soluble lysate from IPTG induced pMAL-MBP-SPP cells generated 4–5 times more NBD-Sph (Fig. 2b,c), suggesting that *L. major* SPP can dephosphorylate S1P into Sph (Fig. 1). The background activity in uninduced cells and cells with empty pMAL-c5x vector may derive from the phosphatase activity in *E. coli* which is known to act on phosphatidic acid, lysophosphatidic acid (LPA), and S1P^37,38^. We were unable to test if *L. major* SPP can degrade dihydrosphingosine-1-phosphate due to the lack of commercially available fluorescently tagged substrate.

**Figure 3:**
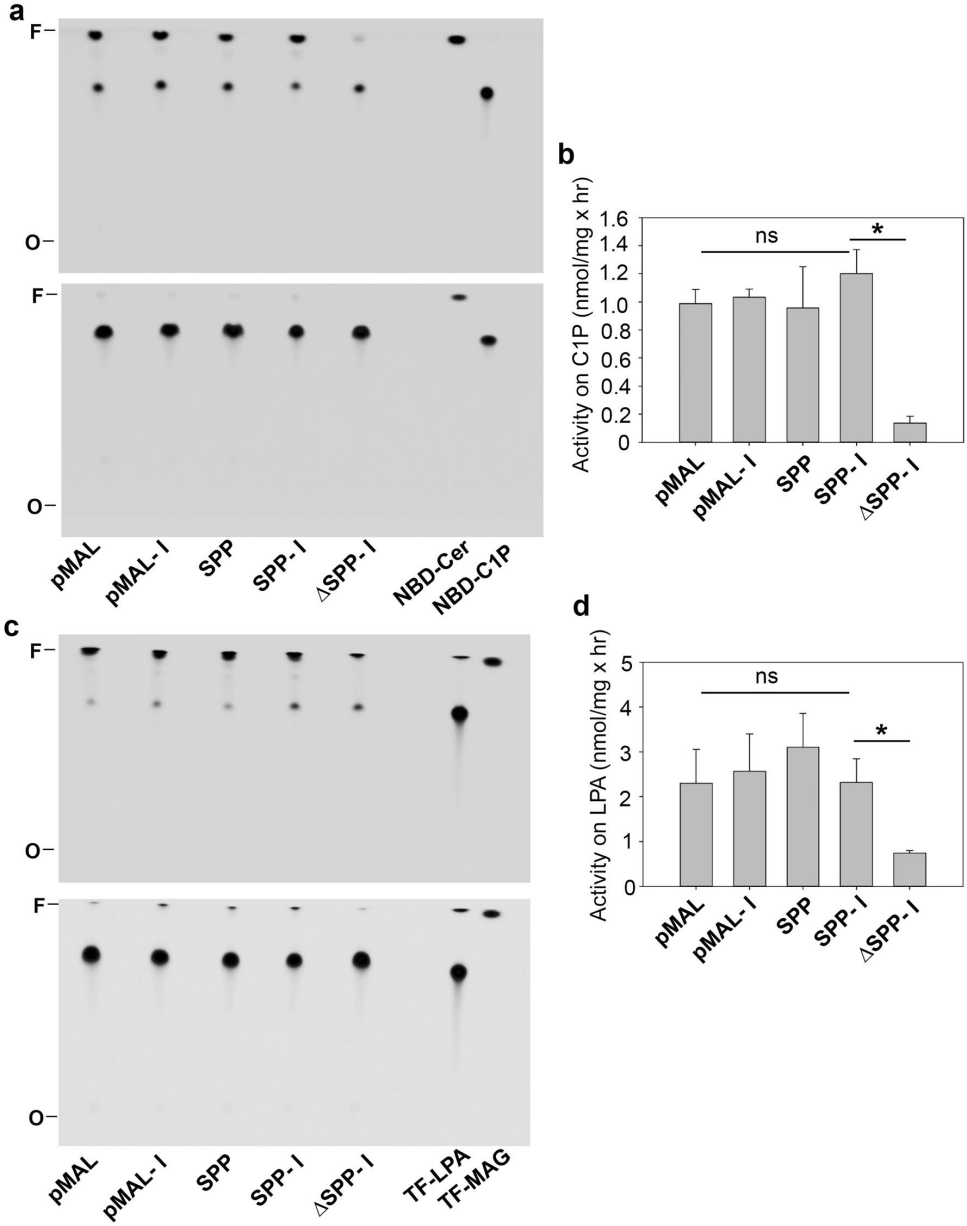
Recombinant *Leishmania major* SPP exhibits no specific activity towards NBD-ceramide-1-phosphate (C1P) or TopFluor-lysophosphatidic acid (TF-LPA). Soluble lysates from *E. coli* were incubated with NBD-C1P (**a**, **b**) or TF-LPA (**c**, **d**) followed by lipid extraction and thin layer chromatography. In (**a**) and (**c**), top panels represent the organic phase of reactions and bottom panels represent the aqueous phase. Pure NBD-C1P, NBD-Cer, TF-LPA, and TF-MAG were included as references. Boiled lysate of induced pMAL-SPP (SPP-I) was used as a control. *F* solvent front, *O* origin. Activities on NBD-C1P and TF-LPA were quantified and plotted in (**b**) and (**d**). *p < 0.05.

To examine whether *L. major* SPP possesses activities towards similar lipid substrates such as ceramide-1-phosphate (C1P) and LPA, we incubated *E. coli* lysates containing MBP-SPP or MBP with a NBD-C1P or C12 TopFluor-LPA (Fig. 3). While all the bacterial lysates had some background activities, no specific activity towards C1P or LPA was detected from MBP-SPP (Fig. 3). Thus, the dephosphorylating activity of *L. major* SPP appears to be specific for S1P.

### Episomal expression and Golgi-localization of GFP-tagged SPP in *L. major*

**Figure 4:**
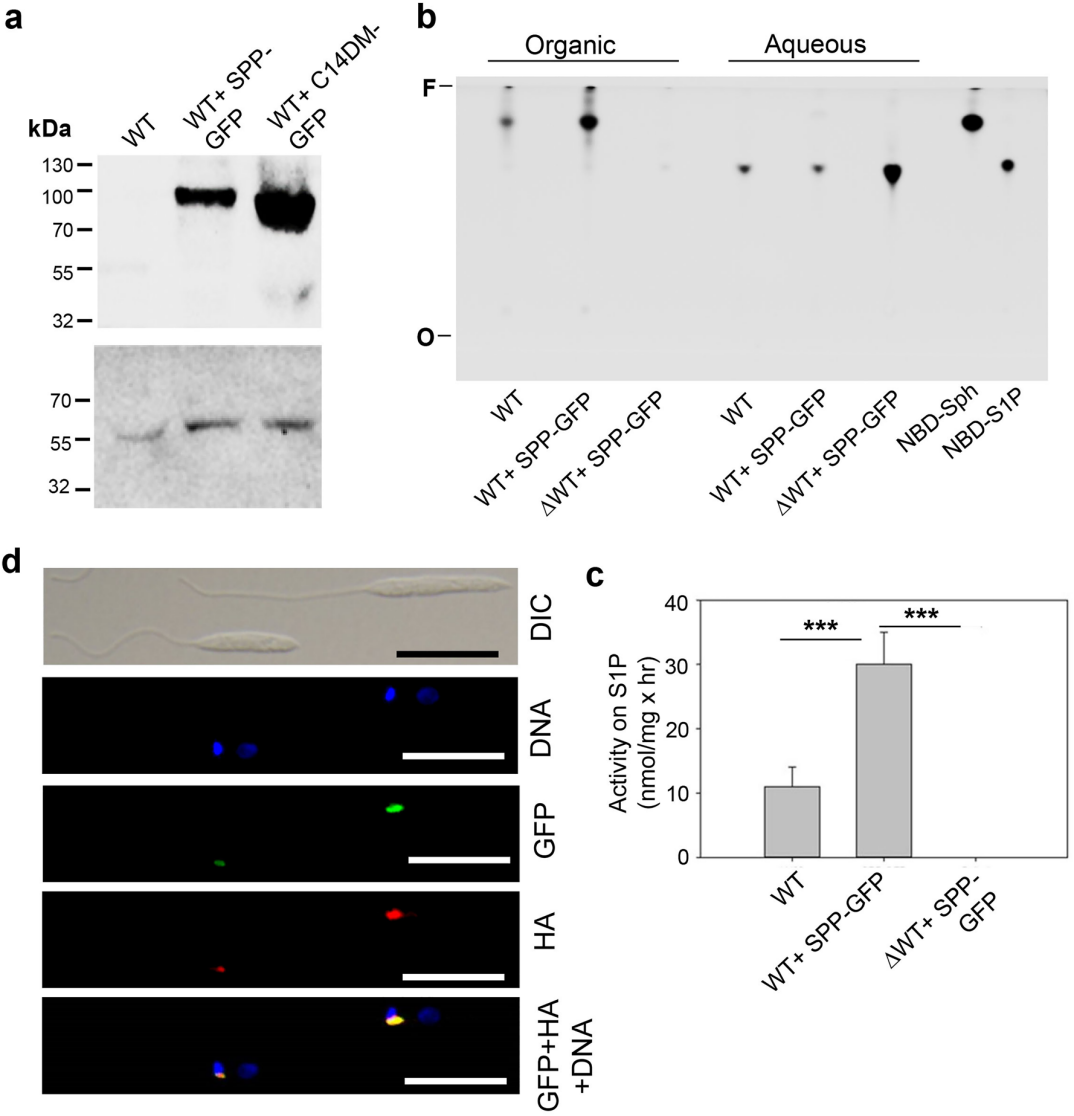
SPP-GFP exhibits activity towards S1P and is localized at the Golgi apparatus in *Leishmania major*. (**a**) Promastigote lysates of WT, WT + SPP-GFP, and WT + C14DM-GFP were probed with an anti-GFP antibody (top) or anti-tubulin antibody (bottom) by western blot. (**b**) Promastigote lysates of WT and WT + SPP-GFP were incubated with NBD-S1P followed by lipid extraction. Both the organic and aqueous fractions were analyzed by thin layer chromatography. Pure NBD-S1P and NBD-Sph were included as references. Boiled lysate of WT + SPP-GFP (WT + SPP-GFP) was used as a control. *F* solvent front, *O* origin. (**c**) Quantitation of the enzymatic conversion of NBD-S1P into NBD-SPH by promastigote lysates. Error bars represent standard deviations from three experiments. **p < 0.01. ***p < 0.001. (**d**) *L. major* promastigotes containing pXG-SPP-GFP and pX63-LPG2-HA (a Golgi marker) were processed for immunofluorescence microscopy using an anti-HA monoclonal antibody. The Pearson’s Correlation Coefficients between GFP and HA, calculated from 50 cells, was 0.82 ± 0.09 (average ± standard deviations). Scale bars: 10 µm. The full-length blots for (**a**) are included in Supplemental Fig. S3.

To determine the localization of SPP in *L. major*, a SPP-GFP fusion gene was cloned into a high copy number expression plasmid to generate pXG1a-SPP-GFP and introduced into WT promastigotes. SPP-GFP or the C-terminal GFP-tagged SPP was detected at the expected size of ~ 98 kDa by western blot (Fig. 4a, WT + C14DM-GFP was used as a control for ~ 82 kDa^39^) (Supplemental Fig. S3). To examine if the fusion protein is active, parasite lysates were incubated with NBD-S1P followed by lipid extraction and TLC analyses of organic and aqueous phases. As shown in Fig. 4b and c, WT promastigotes possessed the ability to convert NBD-S1P to NBD-Sph. Importantly, this activity was nearly three times higher in WT + SPP-GFP promastigotes and completely lacking in boiled lysates. Thus, SPP-GFP retains the expected SPP activity in *L. major*.

With fluorescence microscopy, SPP-GFP was found at a discrete spot adjacent to the kinetoplast of promastigotes resembling the location of Golgi apparatus (Fig. 4d). To confirm this result, we introduced a haemagglutinin (HA)-tagged LPG2, a known Golgi-localized protein^40^, into WT + SPP-GFP promastigotes. Immunofluorescence microscopy revealed a high degree of overlap between SPP-GFP and LPG2-HA (Fig. 4d; the Pearson’s Correlation Coefficient is 0.82 ± 0.09 after analysis by Image J), indicating that SPP is mainly located in the Golgi apparatus in *L. major* promastigotes.

### Homozygous *SPP* knockout cannot be generated even in the presence of episomal* SPP*

**Figure 5:**
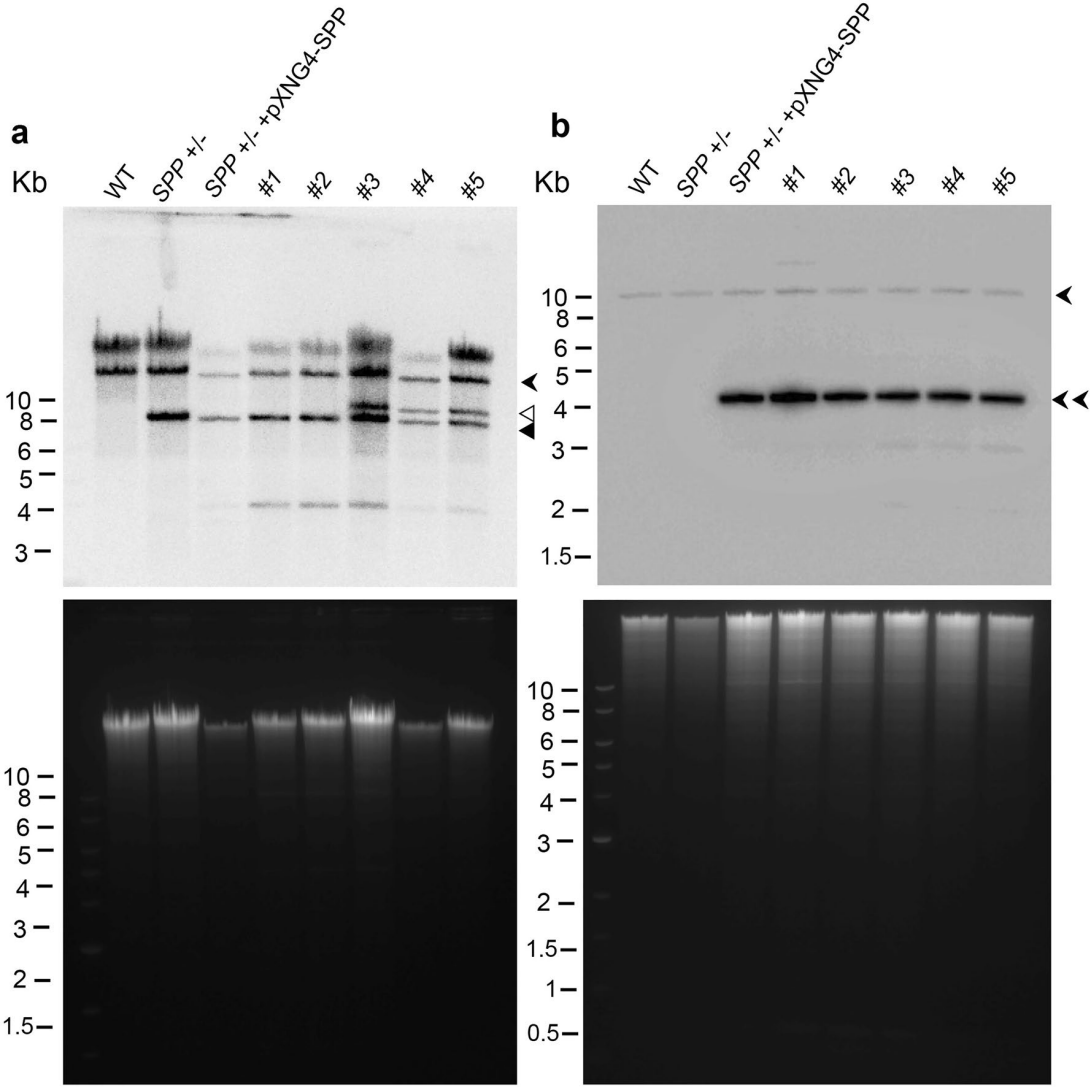
Chromosomal *SPP* is recalcitrant to deletion even in the presence of episomal *SPP*. Genomic DNA samples from WT, *SPP*+/−*, SPP*+/− + pXNG4-SPP and five putative *spp*^−^ + pXNG4-SPP clones (#1–5) were digested with SpeI (**a**) or BamHI (**b**) and subjected to Southern blot analysis using a flanking region probe (**a**) or a *SPP* ORF probe (**b**). In **a** and **b**, top images represent Southern blot results and bottom images represent ethidium bromide staining results. Expected positions for the chromosomal *SPP* ( ), episomal *SPP* (), overexpressed from pXNG4-SPP, a high copy number plasmid), *HYG* (◃) and *BSD* (◂) genes are indicated.

To investigate the roles of *SPP* in *L. major*, we tried to delete the chromosomal alleles of *SPP* using the classical method based on homologous recombination^41^. While the first *SPP* allele could be replaced by the *BSD* resistance marker gene to generate *SPP*+/**−** (the half knockout or heterozygous knockout), repeated attempts to knockout the 2nd and final allele with *HYG* resulted in either no viable cells or cells retaining the *SPP* gene despite showing resistance to blasticidin and hygromycin (Supplemental Figs. S4, S5 and Fig. 5). These findings suggest that SPP is required for survival in *L. major*.

Previous studies by us and other groups have used a plasmid facilitated approach to verify whether a particular gene of interest is essential^42–44^. To apply this strategy, a pXNG4-SPP plasmid was introduced into *SPP*+/**−** followed by attempts to delete the second genomic allele with the *HYG* resistance marker gene (Supplemental Fig. S6). If the resulting chromosomal *SPP*-null mutants could not lose the pXNG4-SPP plasmid via negative selection, then *SPP* is essential^44,45^. To our surprise, even in the presence of the complementing episome, all the clones still retained a chromosomal *SPP* allele, as indicated by Southern blot (Fig. 5a: flanking sequence probe; Fig. 5b: *SPP* coding region probe) and PCR (Supplemental Figs. S7a–c and S8). Several clones showed the expected integration of both *BSD* and *HYG* marker genes in addition to chromosomal *SPP* (#3, #4 and #5 in Fig. 5, and #3 in Supplemental Figs. S7a–c and S8) and episomal *SPP* (high copy number, Fig. 5b), indicating gene amplification. As a technical control, we employed the same approach on the farnesyl diphosphate synthase (FPPS) gene and successfully generated the chromosomal *FPPS*-null mutant in the presence as pXNG4-FPPS as previously described (Supplemental Figs. S7d–f and S9)^43^. Together, these findings argue that unlike other essential genes, chromosomal *SPP* cannot be fully complemented by episomal *SPP*.

### *SPP* overexpression leads to reduced growth and heightened sensitivity to a subset of phosphatase inhibitors

Our previous work on *L. major* SKa reveals the toxic effects of sphingoid base accumulation^25^. Because SPP overexpression may produce elevated level of sphingoid base, we examined whether *SPP*+/**−** + pXNG4-SPP parasites displayed any overt growth defects. As indicated in Fig. 5b, the *SPP* gene copy number in *SPP*+/**−** + pXNG4-SPP was much higher than WT and *SPP*+/**−** cells, which would lead to increased expression of SPP as shown in previous studies on other genes^44,46,47^. We also examined the GFP expression level in *SPP*+/**−** + pXNG4-SPP parasites by flow cytometry. Because GFP and SPP are co-expressed from the same pXNG4-SPP plasmid, the level of GFP reflects the level of SPP^42,48^. As shown in Supplemental Fig. S10, *SPP*+/**−** + pXNG4-SPP had similar GFP levels as WT + SPP-GFP which possessed ~ 3 times more SPP activity than WT parasites (Fig. 4b,c), Thus, in comparison to WT and *SPP*+/−, SPP+/**−** + pXNG4-SPP cells have higher SPP expression levels.

**Figure 6:**
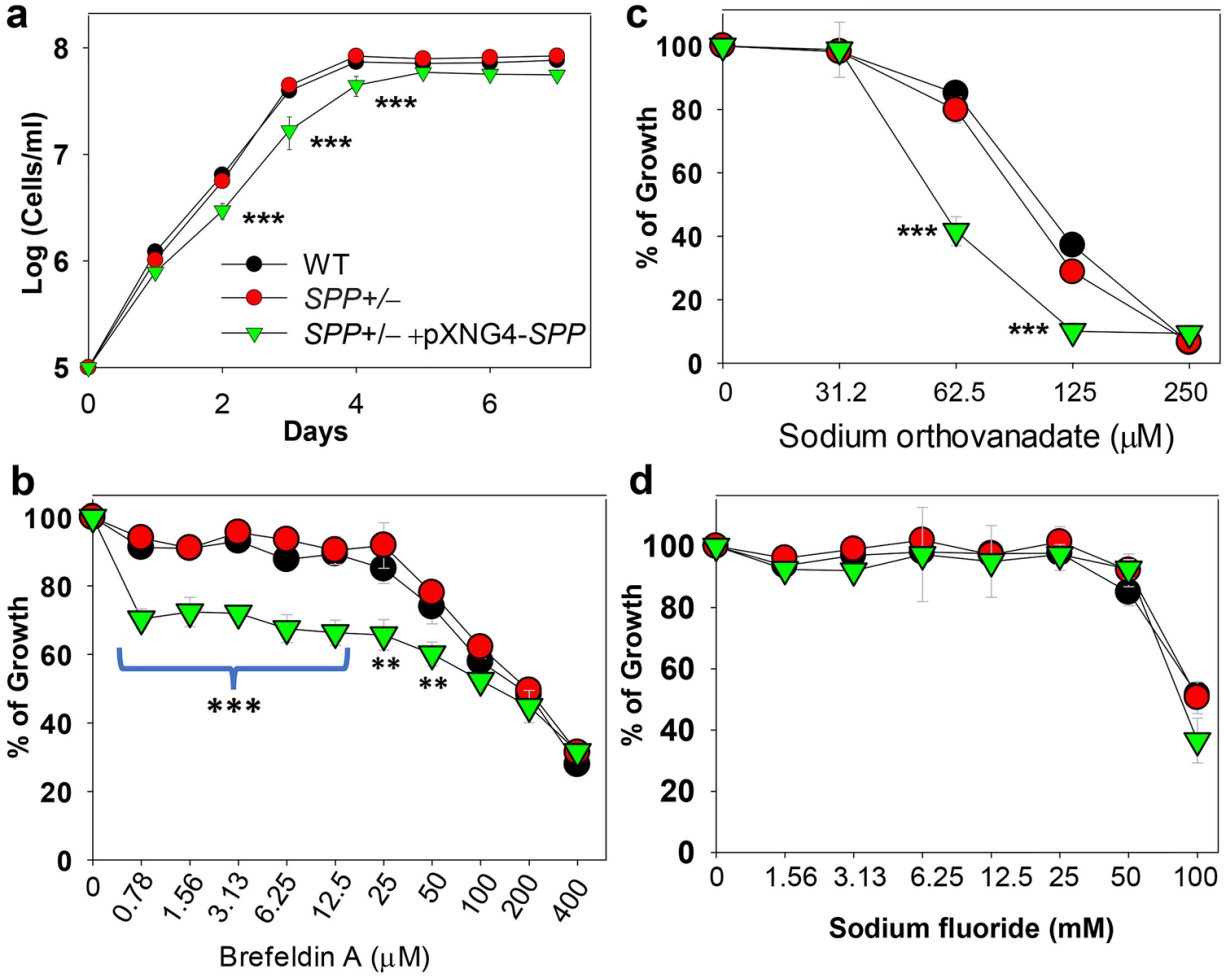
*SPP* overexpression leads to moderate growth defects and increased sensitivity to some phosphatase inhibitors. (**a**) Promastigotes of WT, *SPP*+/−*,* and *SPP*+/− + pXNG4-SPP were cultivated as described in “Methods”. Culture densities were determined daily. In (**b–d**), log phase promastigotes were cultivated in various concentrations of brefeldin A (**b**), sodium orthovanadate (**c**), or sodium fluoride (**d**); culture densities were determined after 48 h; and effects on growth were measured in comparison to cells grown in the absence of inhibitors. Error bars represent standard deviations from four experiments. **p < 0.01; ***p < 0.001.

As indicated in Fig. 6a, these *SPP*+/**−** + pXNG4-*SPP* promastigotes were viable and replicative in culture but showed significant growth delay in comparison to WT and *SPP*+/**−** parasites. When inoculated at 1.0 × 10^5^ cells/ml, it took *SPP*+/**−** + pXNG4-*SPP* five days to reach a maximal density of 5.0–6.0 × 10^7^ cells/ml, whereas WT and *SPP*+/**−** only needed four days to reach 7.1–8.0 × 10^7^ cells/ml (Fig. 6a).

Next, because of its localization, we tested whether SPP overexpression or half knockout affected the Golgi apparatus in *L. major*. Brefeldin A inhibits the GDP/GTP exchange reaction on adenosine ribosylation factors, leading to blockage of intracellular vesicle formation and fragmentation of the Golgi^49,50^. In a 48-h assay, brefeldin A reduced the growth of *SPP*+/**−** + pXNG4-*SPP* by 25% at 0.78 μM whereas nearly 50 μM of brefeldin A was needed to achieve the same level of inhibition for WT and *SPP*+/**−** parasites (Fig. 6b). Thus, the overexpression of SPP (a Golgi-localized protein) makes *Leishmania* hypersensitive to brefeldin A, a Golgi-disrupting chemical.

**Figure 7:**
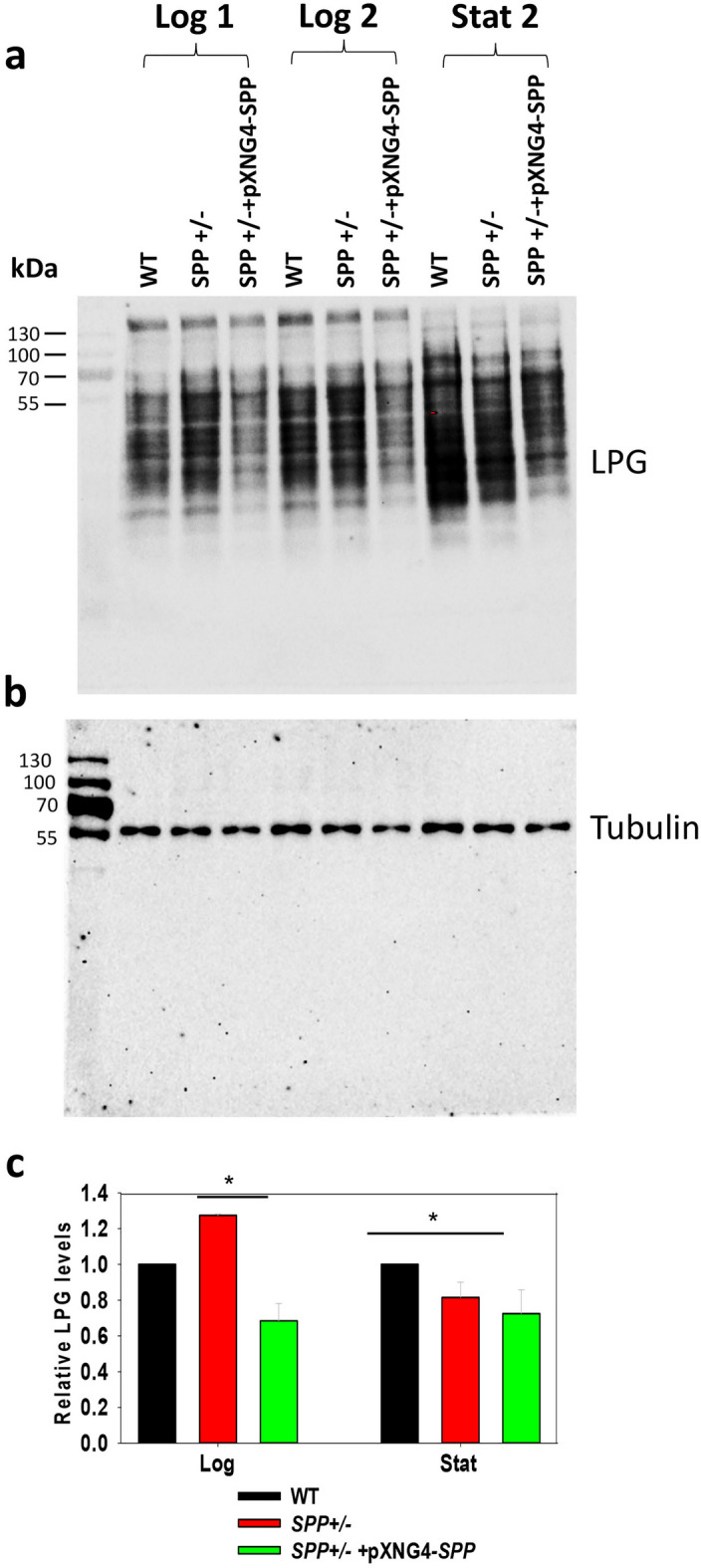
*SPP* overexpression leads to reduced expression of lipophosphoglycan (LPG). Whole cell lysates from log (day 1 and day 2) and stationary phase (day 2) promastigotes were resolved by SDS-PAGE and probed with antibodies against LPG (**a**) or α-tubulin (**b**). The relative level of LPG was determined from three independent experiments (**c**). *p < 0.05.

We also assessed the impact of SPP overexpression and half knockout on the synthesis of lipophosphoglycan (LPG), a large surface glycoconjugate mainly assembled in the Golgi apparatus. Western blot using an anti-LPG side chain Gal residue antibody (WIC79.3)^51^ revealed a 20–30% reduction in the LPG of *SPP*+/**−** + pXNG4-*SPP* during log phase and stationary phase (Fig. 7), suggesting that excessive SPP negatively affected the synthesis and/or trafficking of LPG. No statistically significant difference between WT and *SPP*+/**−** parasites was detected.

Finally, we examined whether SPP overexpression or half knockout altered *L. major*’s sensitivity to general phosphatase inhibitors. Sodium orthovanadate can act as a competitive inhibitor of ATPases, alkaline and acid phosphatases, and protein-phosphotyrosine phosphatases^52,53^. Sodium fluoride is a commonly used general inhibitor for protein phosphoserine and phosphothreonine phosphatases with reported inhibitory effect on mammalian SPP1^28,29,52^. As shown in Fig. 6c, *SPP*+/**−** + pXNG4-*SPP* parasites displayed heightened growth inhibition by sodium orthovanadate, as their EC90, EC50 and EC25 (effective concentrations required to inhibit growth by 90%, 50% and 25% respectively) were 1.7–2.2 times lower than those of WT and *SPP*+/**−**. Meanwhile, we did not detect any significant difference in sensitivity to sodium fluoride (Fig. 6d), suggesting that SPP is not responsive to this inhibitor.

## Discussion

Compared to SK, SPP is relatively understudied. In this report, we verified that *L. major* possesses a functional SPP that can dephosphorylate NBD-labeled S1P but not ceramide-1-phosphate or LPA (Figs. 2 and 3). Such specificity is in line with the SPPs in mammals and yeast^28,30,54^. Despite repeated attempts, we were unable to generate homozygous *SPP* knockout in *L. major*, as clones selected after two rounds of replacement showed gene or chromosomal duplications (Fig. 5 and Supplemental Fig. S5). While the episome facilitated knockout approach has allowed us and others to acquire chromosomal null mutants for several essential genes including *FPPS*, *CEPT* and DHCH1^42,43,45^, we could not fully delete endogenous *SPP* even in the presence of a complementing plasmid (Fig. 5 and Supplemental Figs. S7–S9). These findings suggest that ectopically expressed *SPP* is not equal to endogenous *SPP*, even though it is catalytically active including the GFP-tagged version (Fig. 4). One possibility is that elevated SPP activity from episomal overexpression is harmful which is somewhat supported by the reduced proliferation rate of SPP-over-expressing cells (Fig. 6a). However, the growth defect is relatively modest and seems insufficient to explain our result by itself. Another possibility is that endogenously expressed *SPP* is subjected to essential regulations at the posttranscriptional and/or posttranslational levels and such regulations require the flanking sequences of *SPP* which are absent in the SPP-expression plasmid.

The fact that *L. major* SPP is indispensable makes it distinct from its orthologs in mammals, yeasts and Arabidopsis (SPP is not essential in those eukaryotes)^26–32^. It is possible that S1P accumulation from SPP knockout is deleterious to *L. major* promastigotes. Previous studies on *L. major* SKa suggests that the cellular level of S1P must be carefully controlled for parasite survival in mice, as only the SKa chromosomal knock-in but not episomal overexpression restores the virulence of *ska*^—^ mutants^25^. On the other hand, SKa overexpression (which should result in S1P accumulation in the cytoplasm) does not cause any detectable defects in *L. major* promastigotes^25^. So it is possible that the dephosphorylation of S1P into Sph in specific cellular locations is required to avoid toxicity.

Interestingly, *L. major* SPP is localized in the Golgi apparatus (Fig. 4d) as opposed to ER as observed for its orthologs in mammals, yeasts and *Arabidopsis*^29,31,32^, suggesting that its functions extend beyond bulk lipid synthesis. In *Leishmania*, the other two enzymes directly involved in S1P metabolism, SKa and SPL, are located in the cytoplasm and ER, respectively (Fig. 1)^16,25^. One possibility is that cytoplasmic S1P (produced by SKa) is transported to different cellular compartments to fulfill distinct functions: it can be used to drive ethanolamine phospholipid synthesis in the ER (mediated by SPL), to regulate the function of Golgi apparatus (mediated by SPP), or stay in the cytoplasm to control stress response via unknown mechanisms. Based on this study, the local concentration of S1P or the ratio of S1P:Sph is likely crucial for maintaining Golgi’s function as a platform for the assembly of macromolecules. While we did not detect any defects in the *SPP*+/**−** promastigotes (half knockout), overexpression of *SPP* led to heightened sensitivity to brefeldin A, a blocker of ER-Golgi vesicular trafficking, and sodium orthovanadate, a general phosphatase inhibitor (Fig. 6). *SPP* overexpression also resulted in slower growth rate and reduced synthesis of LPG (Fig. 7), a major glycolipid and virulence factor in *Leishmania*^55^. Future studies will identify the molecular targets of S1P in various organelles and reveal the underlying mechanism of why the SPP expression level is vital.

In mammals, yeasts and Arabidopsis, the catalytic activity of SPP overlaps with those of LPP^9,35,36,54^. Human cells possess two isoforms of LPP, with LPP2 in the plasma membrane and early endosomes and LPP3 in the plasma membrane and the Golgi apparatus^56,57^. Both human LPPs can dephosphorylate S1P, and chemical or genetic depletion of LPP3 can block the production of vesicles from the Golgi^54,56,57^. Genetic knockdown of *LPP2* results in delay of cells reaching S-phase, whereas *LPP2* overexpression leads to premature entry into the S-phase^57^. *L. major* has at least one LPP homolog (from https://tritrypdb.org/tritrypdb/app, gen ID: LmjF.19.1350), raising questions of whether this enzyme can recognize S1P/dihydrosphingosine-1-phosphate as substrates and how its expression is regulated. Given the fact that *SPP* is essential, it appears *LPP* alone is insufficient to fully substitute *SPP*.

In summary, our characterization of SPP along with previous work on SKa indicate that the balance between S1P and Sph is crucial for the function of Golgi apparatus in *Leishmania*. Thus, in addition to providing ethanolamine phosphate for lipid synthesis (mediated by SPL), sphingoid base metabolism regulates vital processes to ensure the viability and fitness of *Leishmania* parasites. Future mechanistic studies will inform the development of novel therapeutics targeting sphingoid base metabolism.

## Methods

### Materials

Omega(7-nitro-2-1,3-benzoxadiazol-4-yl)-D-*erythro*-sphingosine-1-phosphate (C18 NBD-S1P), Omega(7-nitro-2-1,3-benzoxadiazol-4-yl) (2S,3R,4E)-2-aminooctadec-4-ene-1,3-diol (C18 NBD-Sph), 1-[12-[4-(dipyrrometheneboron difluoride)butanoyl]amino]dodecanoyl-2-hydroxy-sn-glycero-3-phosphate (C12 TopFluor-LPA), 1-[11-(dipyrrometheneboron difluoride)undecanoyl]-*rac*-glycerol (C11 TopFluor-MAG), and N-[6-[(7-nitro-2-1,3-benzoxadiazol-4-yl)amino]hexanoyl]-D-*erythro*-sphingosine (C6 NBD-Cer) were purchased from Avanti Polar Lipids. N-[6-[(7-nitro-2-1,3-benzoxadiazol-4-yl)amino]hexanoyl]-D-*erythro*-sphingosine-1-phosphate (C6 NBD-C1P) was purchased from Echelon Biosciences. 500 µM stocks of NBD-S1P, NBD-C1P, and TopFluor LPA were prepared in methanol and stored at – 20 °C. Oligonucleotide primers (summarized in Supplementary Table S1) were synthesized by Integrated DNA Technologies and all other materials were purchased from Thermo Fisher Scientific unless otherwise specified.

### Molecular constructs

The open reading frame (ORF) of *L. major SPP* (tritrydb: LmjF.32.2290; Genbank accession number: Q4Q546) was amplified via PCR using primers #915 and #472 from *L. major* genomic DNA and cloned into pMAL-c5x (purchased from New England Biolabs) or pXNG4^42^ vectors to generate pMAL-MBP-SPP or pXNG4-SPP, respectively. Primers #916 and #505 were used to amplify a modified *SPP* ORF without stop codon which was cloned into a pXG1a-‘GFP vector to generate pXG1a-SPP-GFP. To acquire knockout constructs, the *SPP* upstream and downstream flanking sequences (~ 2 Kb each) were amplified via PCR and cloned into a pUC18 vector before genes conferring blasticidin or hygromycin resistance were inserted between these flanking sequences to generate pUC18-5′ UTR-*BSD*-3′ UTR SPP or pUC18-5′ UTR-*HYG*-3′ UTR SPP. All molecular constructs were verified by restriction enzyme digestion and DNA sequencing.

### *Leishmania* culture and genetic manipulation

*Leishmania major* Friedelin V1 (MHOM/IL/80/FN) promastigotes were cultivated at 27 °C in M199 media supplemented with 10% heat inactivated fetal bovine serum and other additives as described previously^58^. To monitor growth, culture densities were determined daily using a Neubauer hemocytometer. To measure sensitivity to phosphatase inhibitors, log phase promastigotes were inoculated in complete M199 media at 2.0 × 10^5^ cells/ml in the absence or presence of brefeldin A (0–400 μM), sodium orthovanadate (0–250 μM), or sodium fluoride (0–100 mM); and culture densities were determined after 48 h.

To delete the first chromosomal *SPP* allele, a DNA fragment conferring resistance to blasticidin was obtained by restriction enzyme digestion of pUC18-5′ UTR-*BSD*-3′ UTR SPP and introduced into *L. major* wild type (WT) promastigotes via electroporation. The resulting *SPP*+/− parasites were confirmed by Southern blot using probes corresponding to the open reading frame and an upstream flanking sequence of *SPP*. To delete the second *SPP* allele, a DNA fragment conferring resistance to hygromycin (derived from pUC18-5′ UTR-*HYG*-3′ UTR SPP) was introduced into *SPP*+/− parasites and transfectants showing resistance to blasticidin and hygromycin were analyzed by Southern blot.

To generate chromosomal-null *SPP* mutants in the presence of episomal genes, *SPP*+/− parasites were transfected with pXNG4-SPP, followed by attempts to delete the second chromosomal *SPP* allele using a DNA fragment conferring resistance to hygromycin. To study the localization of SPP, *L. major* WT promastigotes were transfected with pX63HYG-LPG2-HA^40^ and pXG1a-SPP-GFP cells. Generation of *FPPS* transgenic lines was performed as previously described^43^. Transfectants were selected and maintained using blasticidin (10 µg/ml, Corning), hygromycin (50 µg/ml, InvivoGen), G418 (100 µg/ml, Corning), puromycin (20 µg/ml, MP Biomedical), or nourseothricin (100 µg/ml, Gold Biotechnology) as needed. Genetic manipulations of *L. major* cells were validated by Southern blot or PCR as previously described^43^.

### SPP activity assay

*Escherichia coli* BL21(DE3) cells containing pMAL-SPP or pMAL-c5x were cultivated in LB media in the presence of 100 µg/ml of ampicillin at 37 °C until reaching an OD_600_ of 0.6–0.8. Cells were then induced with 0.3 mM of IPTG at room temperature for 4–8 h and collected by centrifugation. Bacterial pellets were resuspended in 200 mM of Tris–HCl (pH 7.0), 200 mM of NaCl, 1 mM of EDTA (pH 8.0), 1 mM of dithiothreitol, and 1 × protease inhibitors (Roche), followed by sonication at 4 °C (3 × 30 s with 1 min in between). The resulting whole cell lysates were centrifuged at 14,000×*g* for 20 min at 4 °C to generate soluble lysates. Protein concentrations were determined using a BCA assay kit (Roche). Aliquots of whole cell lysate and soluble lysate were processed for activity assay as described below. Heat inactivated soluble lysate was prepared by heating lysate at 100 °C for 7 min.

The SPP activity assay was developed based on a previous report^59^. Briefly, 500 µl of soluble lysate containing ~ 1 mg of protein was incubated in the presence of 5 µM of NBD-S1P, NBD-C1P, or TopFluor LPA at 37 °C for 1 h. Samples were then subjected to Bligh-Dyer extraction^60^ to separate organic and aqueous fractions which were air dried and dissolved in 20 µl of chloroform:methanol (1:2). These fractions were loaded on alumina-backed silica gel plates and analyzed by thin layer chromatography (TLC) using a mobile phase of 1-butanol:glacial acetic acid:water 3:1:1. Plates were air dried, and signals were detected with a Bio-Rad Chemidoc MP Imager. Activities were quantified based on the amount of product, the amount of protein and the length of time.

To detect SPP activity in *Leishmania*, promastigotes of WT or WT + pXG1a-SPP-GFP were cultivated to 8–9 × 10^6^ cells/ml and collected by centrifugation. Parasite lysates were prepared and SPP activity were determined as described above for bacterial samples.

### Fluorescence microscopy and western blot

Promastigotes of WT + pX63-LPG2-HA + pXG1a-SPP-GFP were grown to 4–5 × 10^6^ cells/ml, collected by centrifugation and washed with 1 X PBS before fixation with 3.75% formalin and mounted onto poly-lysine-treated coverslips. Cells were then processed for immunofluorescence microscopy as previously described using a mouse anti-HA monoclonal antibody (Sigma, 1:700) followed by a goat anti-mouse IgG conjugated with Texas Red (Life Technologies, 1:700). After staining with 3 µg/ml of HOESCHT 33342, cells were examined using an Olympus DX51 fluorescence microscope. The overlap between LPG2-HA and SPP-GFP was determined using JaCOP ImageJ analysis^61^.

To verify the expression of SPP-GFP, promastigote lysates were probed with a rabbit anti-GFP antiserum (Abcam, 1:1000) followed by a goat anti-rabbit IgG-HRP (1:2000). To detect lipophosphoglycan (LPG), a monoclonal antibody WIC79.3 (1:1000) was used^51^. As a loading control, lysates were probed with a mouse anti-alpha tubulin antibody (Invitrogen, 1:4000) followed by goat anti-mouse IgG-HRP (1:2000). The presence of MBP-SPP in bacterial lysate was verified by western blot using an anti-MBP monoclonal antibody (New England Biolabs) as the primary antibody (1:5000) and a goat anti-mouse IgG HRP-conjugated antibody (1:4000) as the secondary antibody, respectively. Western blots were visualized and quantified using a FluorChem E Imager (ProteinSimple).

### Statistical analyses

Unless otherwise specified, all experiments were repeated 3–4 times and data are represented as means ± standard deviations. Statistical significance was determined by unpaired Student’s *t* test (for two groups) or one-way ANOVA (for three or more groups) using Sigmaplot 13.0 (Systat Software Inc, San Jose, CA). *P* values indicating statistical significance were grouped into values of < 0.05 (*), < 0.01 (**), and < 0.001 (***).

## Supplementary Information

Supplementary Information.

## Data Availability

The DNA/protein sequences of *L. major* SPP analyzed during the current study are available in the Tritrypdb repository (https://tritrypdb.org/tritrypdb/app, gene ID: LmjF.32.2290) and the Genbank (accession number: Q4Q546). All data and materials from this study may be obtained from the corresponding author upon written request with the stipulation that they will be used for research purpose only and any work derived from the data and materials will cite the source.
