# Supplementary material for: The Golgi-localized sphingosine-1-phosphate phosphatase is indispensable for Leishmania major
Source: Sci Rep. 2022 Sep 26;12:16064. doi: 10.1038/s41598-022-20249-w (PMC9513092; doi:10.1038/s41598-022-20249-w)
Supplement: Supplementary file 1 — Supplementary Information. [file 41598_2022_20249_MOESM1_ESM.pdf]

The Golgi-localized Sphingosine-1-Phosphate Phosphatase is Indispensable for *Leishmania major*

Brian Okundaye, Neha Biyani, Samrat Moitra, and Kai Zhang

Supplementary figures and tables

Supplementary Table S1. Oligonucleotide primers used in this study

Primer number	Primer Sequence	Purpose
915	CGTACGGATCCATGTCAACAGCCACAC ACCCAATG	Forward primer to clone the full length *SPP* ORF (BamHI)
472	CCTCGCGGATCCTCACTCTGGCGACG GTG	Reverse primer to clone the full length *SPP* ORF (BamHI)
916	CCTCGCCCGGGATGTCAACAGCCACAC ACCCAATG	Forward primer to clone the full length *SPP* ORF (XmaI)
505	CCTCCGGATATCCTCTGGCGACGGTGG TGGTG	Reverse Primer to clone *SPP* without the stop codon (ECORV)
564	GCGAGGggtaccACATCCCTTCACATTCGT CG	Forward primer to amplify a 2 Kb upstream region of *SPP* (KpnI)
565	GCGAGGactagtTGGGTGTGTGGCTGTTG AC	Reverse primer to amplify a 2 Kb upstream region of *SPP* (SpeI)
566	GCGAGGactagtGGCTAGagatctACTACGAA GGAGAAGGAGAGAAG	Forward primer to amplify a 2 Kb downstream region of *SPP* (SpeI+BglII)
567	CCTCGCggatccATACTCAGTTAACGCTCG TTTTCG	Reverse primer to amplify a 2 Kb downstream region of *SPP* (BamHI)

```
Sc SPP    ------------------------------------------------------------    0
Lm SPP    MSTATHPMFVVTSKPASHFVGPSSAQDFTPVPSPPLTTLASTRRDAVREGVAVSATCTDD    60
Hs SPP2   -----------------------------------MAELL--------------------    5
Mm SPP    -----------------------------------MAELL--------------------    5

Sc SPP    MVDGLNTSNIRK------------------------------------------RARTLS    18
Lm SPP    TTNGIGNGGIAGVAPCNGNNSGWPYPRGGGNPAANGTASGKSKPISLGIVLKGPNREGES    120
Hs SPP2   --RSLQDSQL--VARFQRRCGLFPAPDEGP--RENGA-----------------------    36
Mm SPP2   --RSLRDSQL--VARFQRRCGLFPAREASG------------------------------    31
             .:  . :

Sc SPP    NPNDFQEPNYLLD---------------------PGNHPSDHFR------TRMSKFRF-    49
Lm SPP    NPSDFAENSDSLGTDAPQVGDTVESAIKVPMTVVTPAAVSSDVVASPLTVHENLVQWRPP    180
Hs SPP2   DP--------------------TERAARVPGVEHLPAANGKGGEA-PA---NGLRRAAAP    72
Mm SPP2   ------------------------------------------------------------    31

Sc SPP    ----NIREKL-LVFTNNQSFTLSRWQKKYR------SAFNDLYFTYTSLMGSHTFYVLCL    98
Lm SPP    QRAVVFREWYISCFCNEQQ---VWWIKKAQRIGGPLQPLLVPYFQLWSFTGEAEFYILFI    237
Hs SPP2   -------EAYVQKYVVK-------------------NYFYYYLFQFSAALGQEVFYITFL    106
Mm SPP2   -----------EEHVVK-------------------NYFYYYLFRFSAALGQEVFYITFL    61
                       .  :                   . :    *   :  *.  **:  :

Sc SPP    PMPVWFGYFETTKDMVYILGYSIYLSGFFKDYWCLPRPRAPPLHRITLSEYTTKEYGAPS    158
Lm SPP    PTTAWLGAPLSSVQMASMLWVGQYVTSTMKDAFCCPRPPCPPLQLHGKRDAHDNEYGFPS    297
Hs SPP2   PFTHWNIDPYLSRRLIIIWVLVMYIGQVAKDVLKWPRPSSPPVVKLEKR--LIAEYGMPS    164
Mm SPP2   PFTHWNIDPNLSRRLVVIWVLVMYIGQVAKDILKWPRPSFPPVVRLEKR--IIAEYGMPS    119
          *   *      :  :  :     *:    **    ***  **:           *** **

Sc SPP    SHTANATGVSLLFLYNIWRMQESSVMVQLLLSCVVLFYYMTLVFGRIYCGMHGILDLVSG    218
Lm SPP    THSCQSGVFSFLLYCQLVHAFPDHAFLCW---LAAVCYFAHVSFSRIYLGMHWVGDLIGG    354
Hs SPP2   THAMAATAIAFTLLISTMDRYQYPFVLGL---VMAVVFSTLVCLSRLYTGMHTVLDVLGG    221
Mm SPP2   THAMAATAISFTLLISTMDRYQYPFILGL---MMAVVFSTLVCLSRLYTGMHTVLDILGG    176
          :*:  :  .:: :  .         .:       .: :   : :.*:* *** : *::.*

Sc SPP    GLIGIVCFIVRMYFK--YRFPG-LRIEEHWWFPLFSVGWGLLLLFKHVKPVDECPCFQDS    275
Lm SPP    GVVAFLTVLSHVAFLDGWEASILQRADTPWWAYLLLYVTVHLLSMAHATPHDPCPCYIDS    414
Hs SPP2   VLITALLIVL---TYPAWTFIDCLDSASPLFPVCVIVVPF-FLCYNYPVSDYYSPTRADT    277
Mm SPP2   VLITAVLIAL---TYPAWTLIDSLDSASPLFPVCVIVVPF-LLCYNYPVSDYYSPTRADT    232
           ::  : .         :            :   .      :*   :      .*   *:

Sc SPP    VAFMGVVSGIECCDWLGKVFGVTLVYNLEPNC------------GWRLT--LARLLVGLP    321
Lm SPP    LRFTGAVMGSAIGLWRFYAIYGTLAARPQSDHMLDVVFSFSFL---------MQWVVCMA    465
Hs SPP2   TTILAAGAGVTIGFWINHFF--QLVSKPAES--LPVIQNIPPLTTYMLVLGLTKFAVGIV    333
Mm SPP2   TTIVAAGAGVTLGFWINHFF--QLVSKPTPS--LPVIQNIPPLTTDMLVLGLTKFMVGIM    288
            : ..  *     *    :   *. .   .                      :  * :

Sc SPP    CVVIWKYVISKPMIYTLLIKVFHLKDDRNVAARKRLEATHKEGASKYECPLYIGEPKIDI    381
Lm SPP    VVFASRE-LSLLIAAVVLKAVFKFL-SGECAPRLPKSLRQ---------P-YLAMAKVVG    513
Hs SPP2   LILLVRQ-LVQNLSLQVLYSWFKVV-TRNKEARRRLEIEV---------P-YKFV----T    377
Mm SPP2   LILLVRQ-LVQKLSLQVLFSWFKVV-TRNKEARRRLEIEV---------P-YKFV----T    332
           :.  :  :   :   :*   *:.    :   *   .            * *

Sc SPP    LGRFIIYAGVPFTVVMCSPVLFSLLNIA--------------------------------    409
Lm SPP    LTTLGNERGSRSCIPVTANGSLPMQSDRFNGGFGQDEKAAAAAADGKNALNSRSNTETVE    573
Hs SPP2   YTSVGI----------CATTFVPML-HRFLGLP---------------------------    399
Mm SPP2   YTSVGI----------CATTFVPML-HRFLGLL---------------------------    354
             .             :   . :

Sc SPP    ------------------------------------------------------------    409
Lm SPP    ELDGYLNSQQVWSLRTHRHWWLWDVHGRTVSYAVMGFVATFVCQVLLRETFGVGQELTAL    633
Hs SPP2   ------------------------------------------------------------    399
Mm SPP2   ------------------------------------------------------------    354

Sc SPP    -------------    409
Lm SPP    AAHRPWSPPPSPE    646
Hs SPP2   -------------    399
Mm SPP2   -------------    354
```

Figure S1. Alignment of SPPs from yeast, *L. major*, human and mouse using the CLUSTAL Omega Alignment. Genbank accession numbers for *Saccharomyces cerevisiae* SPP, *L. major* SPP, *Homo sapiens* SPP2, and *Mus musculus* SPP2 are P47013, Q4Q546, Q8IWX5 and Q810K3, respectively. Asterisks (*) represent fully conserved residues; colons (:) represent strongly similar residues; and periods (.) represent weakly similar residues.

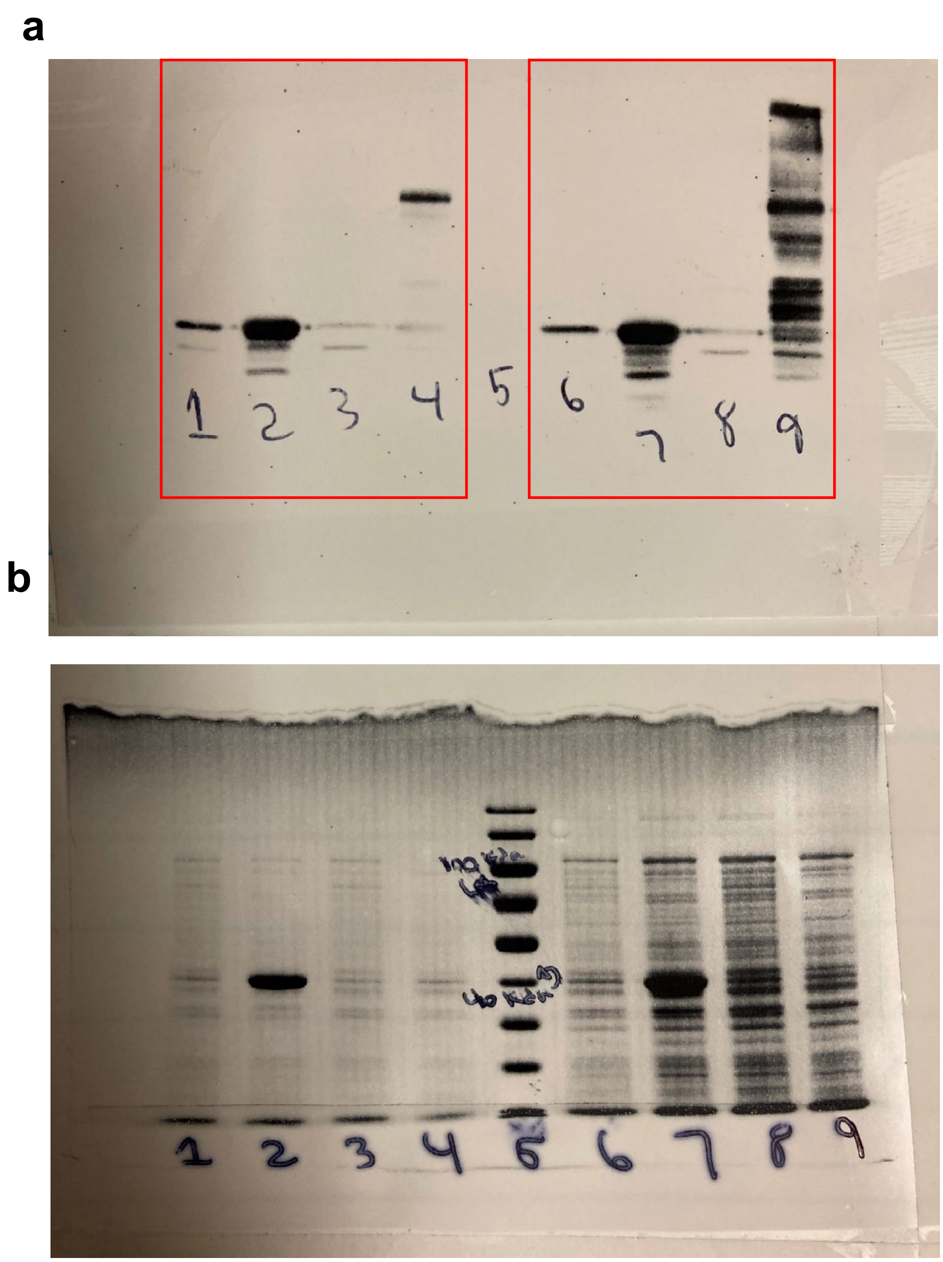

Figure S2. The original, full-length western blots of Fig. 2a. *E. coli* lysates (1-4: soluble fractions; 6-9: whole cell lysate) were resolved by SDS-PAGE followed by western blot analysis using an anti-MBP antibody (**a**) or Coomassie blue staining (**b**). 1, 6: pMAL-c5x vector (pMAL); 2, 7: pMAL-c5x vector with IPTG induction; 3, 8: pMAL-MBP-SPP (SPP); 4, 9: pMAL-MBP-SPP (SPP) with IPTG induction; 5: protein size ladder. The red boxes mark the cropped portions shown in Fig. 2a.

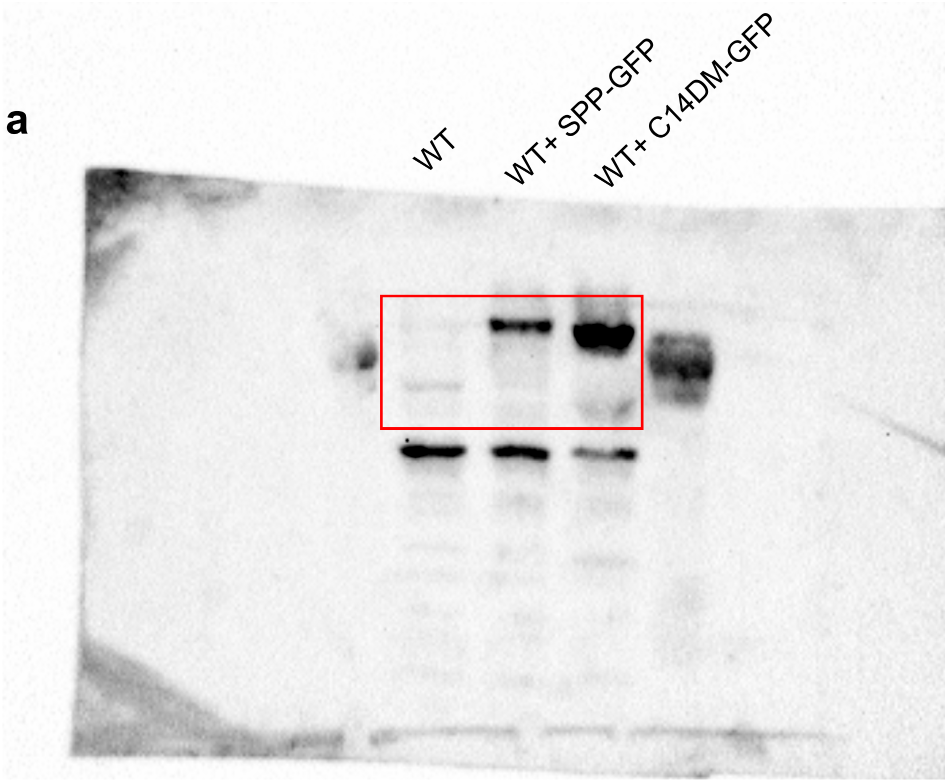

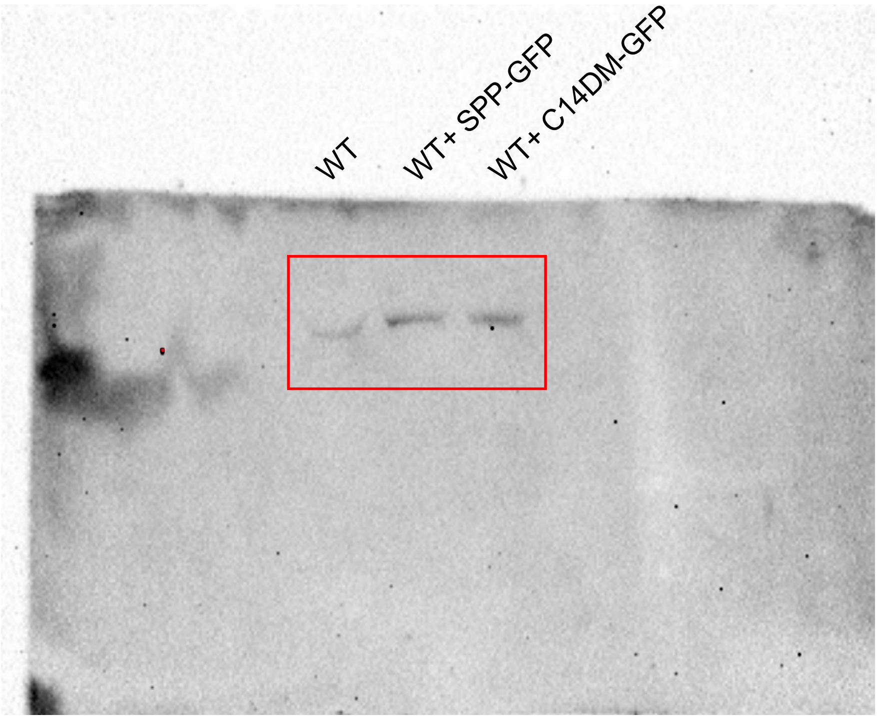

Figure S3. The original, full-length western blots of Fig. 4a. Promastigote lysates of WT, WT+SPP-GFP and WT+C14DM-GFP were probed with an anti-GFP antibody (**a**) or anti-tubulin antibody (**b**) by western blot. The red boxes mark the cropped portions shown in Fig. 4a.

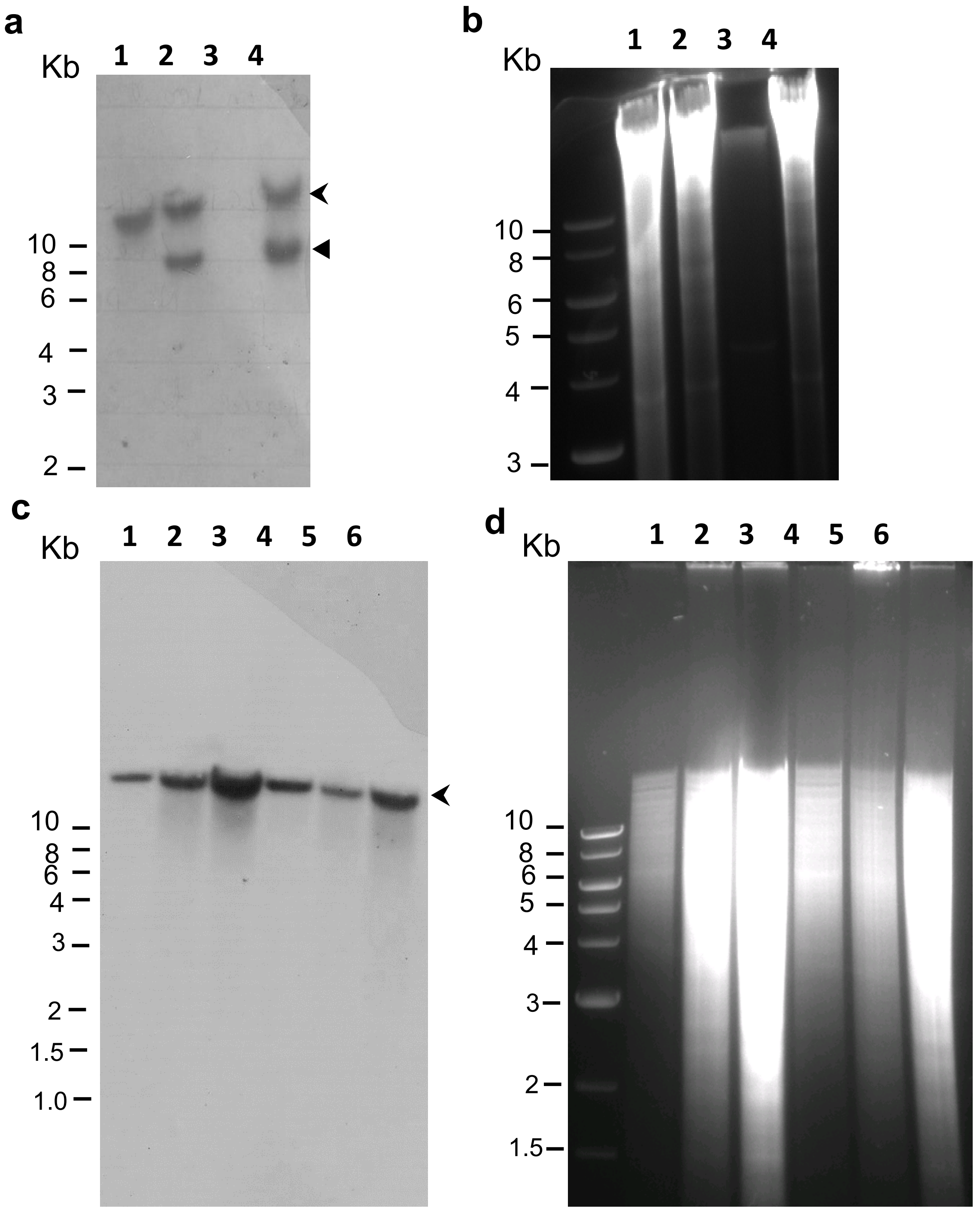

Figure S4. Southern blot analysis of attempted deletion of chromosomal *SPP* in the absence of episomal *SPP*. (**a**-**b**) Genomic DNA samples from WT (lane 1) and three *SPP*+/- clones (lane 2-4 respectively; lane 3 was under-loaded) were digested with SpeI and subjected to Southern blot analysis using a *SPP* flanking region probe (**a**: autoradiograph, **b**: ethidium bromide staining). (**c**-**d**) Genomic DNA samples from WT (lane 1) and five putative spp^{-} clones (lane 2-6) were digested with XhoI and subjected to Southern blot using a *SPP* ORF probe (**c**: autoradiograph, **d**: ethidium bromide staining). Expected positions for the chromosomal *SPP* (❮) and *BSD* (◀) genes are indicated.

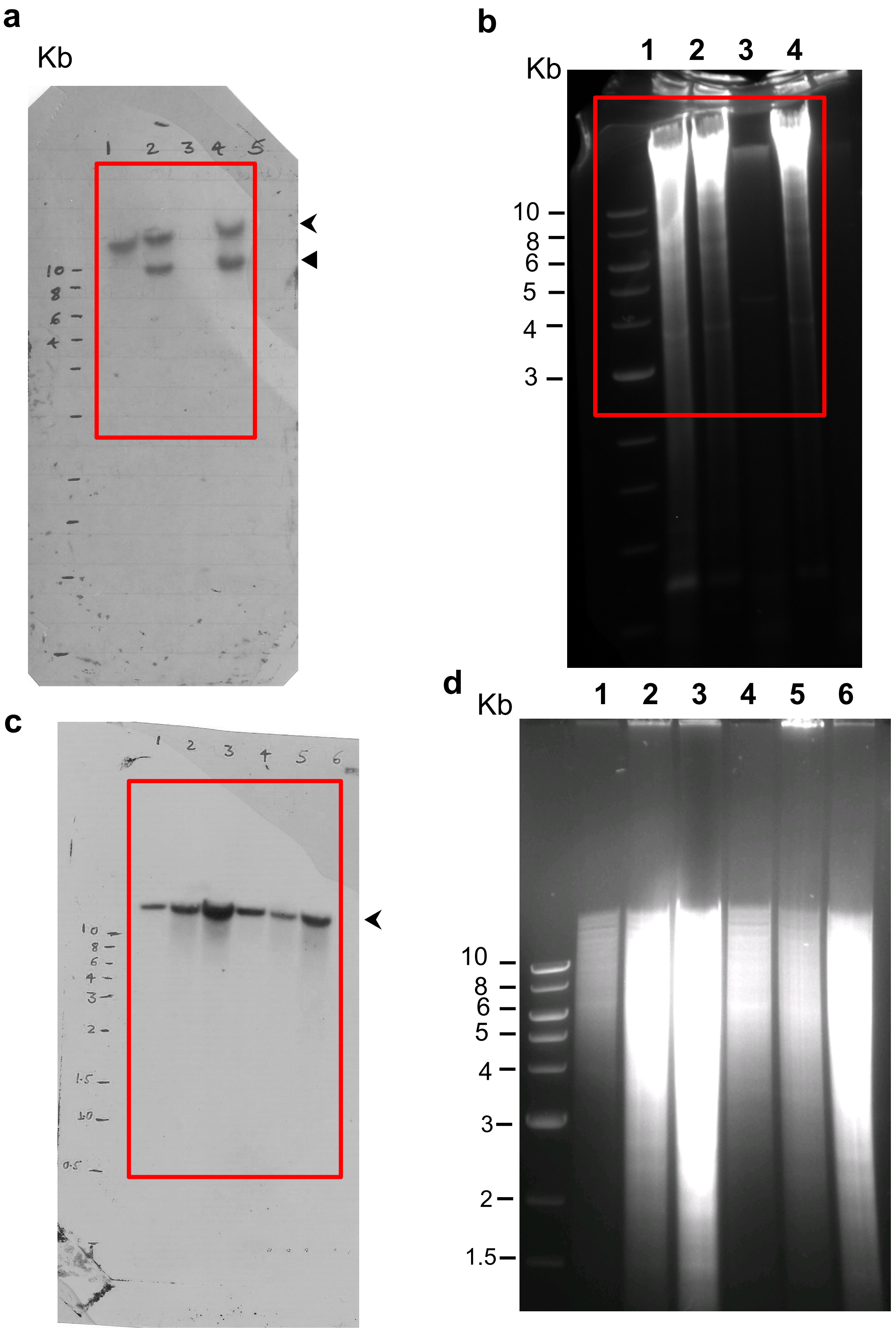

Figure S5. The original, full-length Southern blots of Fig. S4. The red boxes mark the cropped portions shown in Fig. S4.

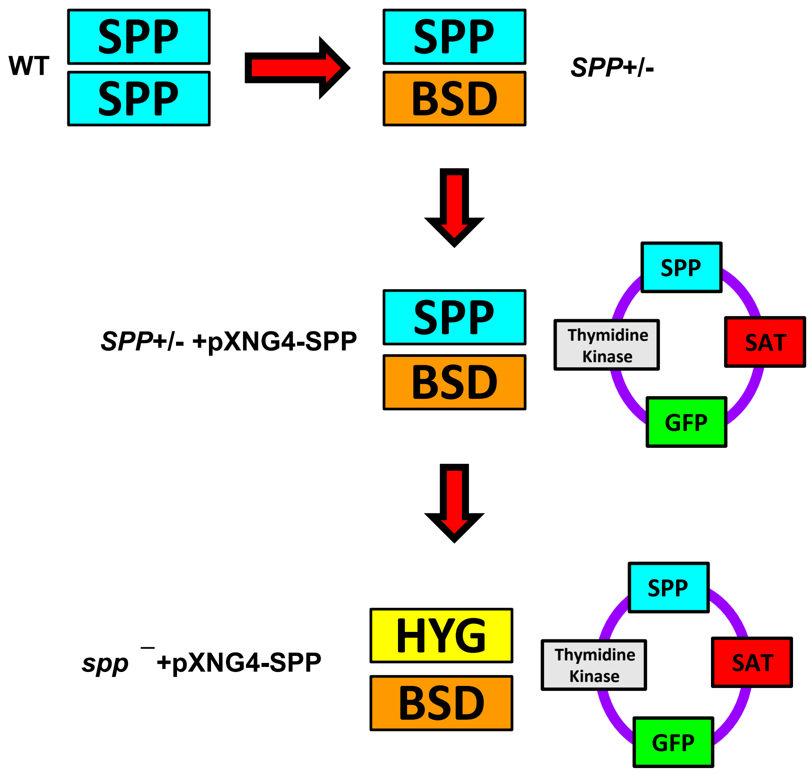

Figure S6. Schematic illustration of the episome assisted knockout strategy for *L. major SPP*. One of the two chromosomal *SPP* alleles in wild type (WT) *L. major* was replaced by *BSD* to generate the heterozygous *SPP* knockout (*SPP*+/-). A pXNG4-*SPP* plasmid was introduced into *SPP*+/- to generate *SPP*+/- +pXNG4-*SPP*. The second chromosomal *SPP* allele was to be replaced by *HYG* (not feasible in this study).

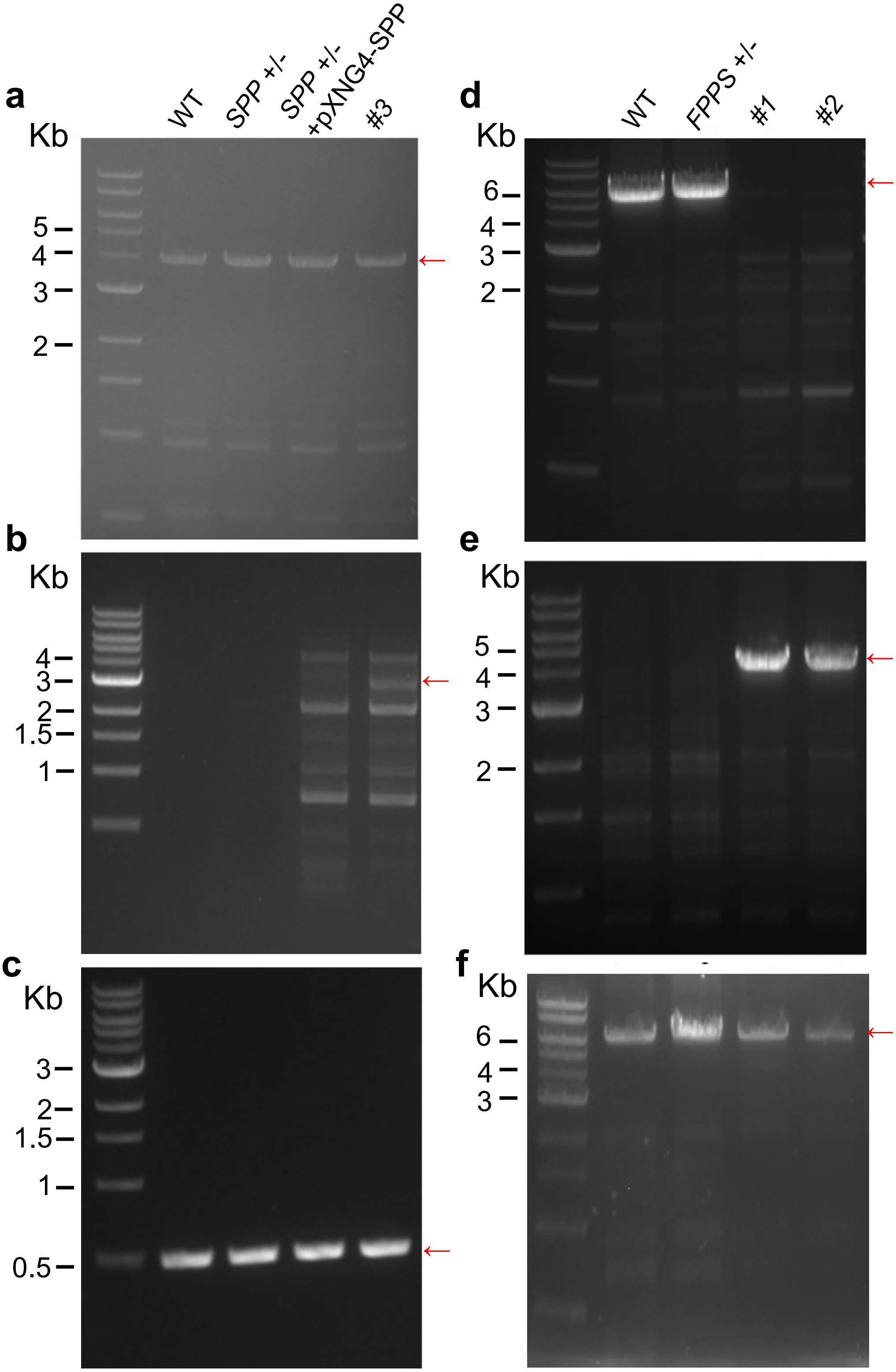

Figure S7. Attempts to delete *L. major SPP* or *FPPS* in the presence of episomal complementing genes. Genetic manipulations of *SPP* or *FPPS* were performed as illustrated in Fig. S5 and results were examined by PCR. In a-c, genomic DNA samples from WT, *SPP*+/-, *SPP*+/- +pXNG4-SPP, and a putative *spp*$^{-}$ +pXNG4-SPP clone #3 were used as templates. In d-f, genomic DNA samples from WT, *FPPS*+/-, and two *fpps*$^{-}$ +pXNG4-FPPS clones were used as templates. Primers were designed to amplify the chromosomal *SPP* and *FPPS* alleles (a and d, respectively), the *HYG* resistance gene used to replace the second chromosomal allele of *SPP* and *FPPS* (b and e, respectively), or control regions (c: a 500-bp fragment of the dihydroceramide synthase ORF; and f: a 6000-bp region including the SPP ORF plus ~2 Kb of upstream and downstream flanking sequences). Red arrows indicate the expected sizes of amplicons.

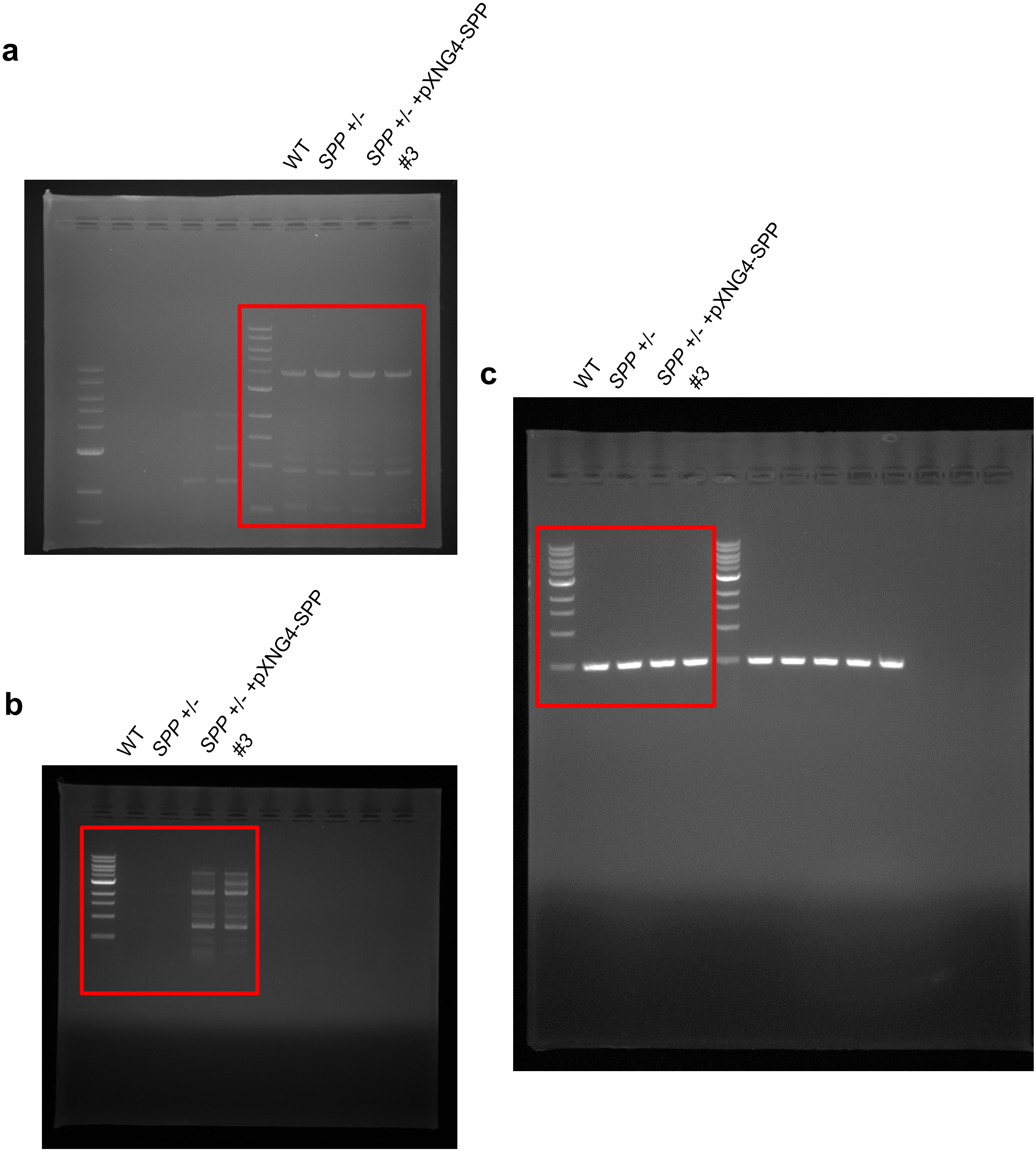

Figure S8. The original, full-length DNA gel images of Fig. S7a-c. The red boxes mark the cropped portions shown in Fig. S7a-c.

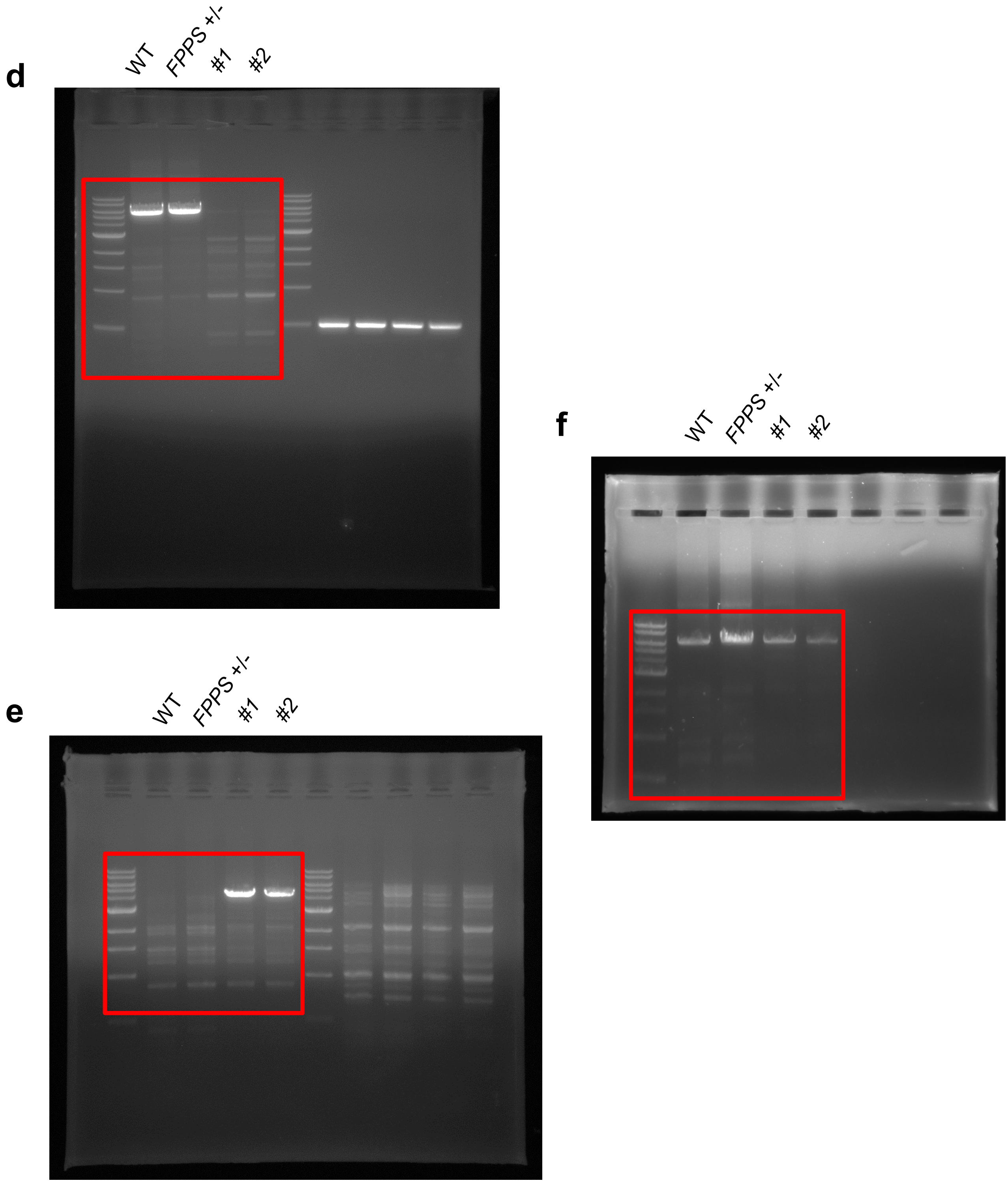

Figure S9. The original, full-length DNA gel images of Fig. S7d-f. The red boxes mark the cropped portions shown in Fig. S7d-f.

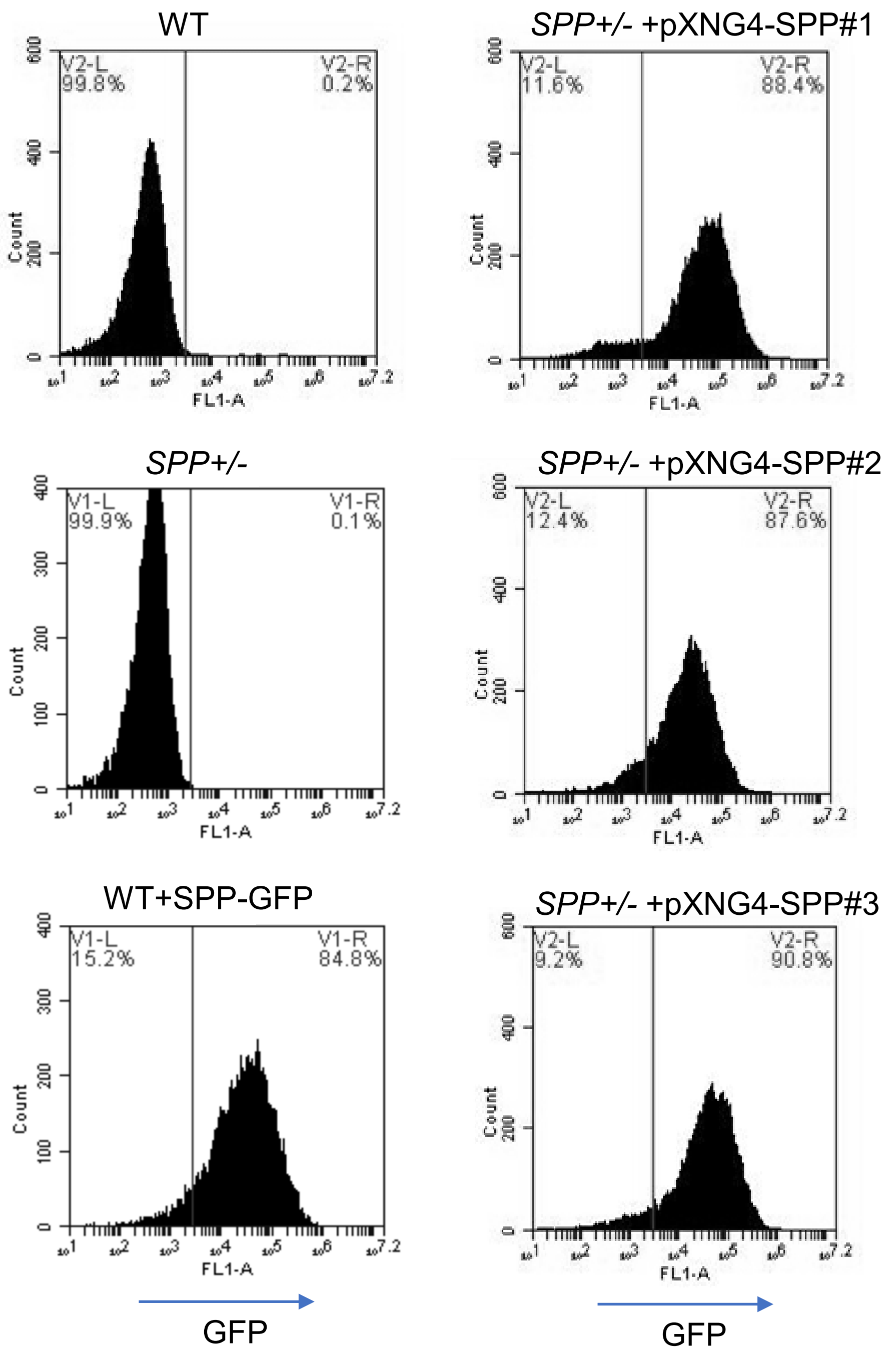

Figure S10. Flow cytometry analysis of SPP+/- +pXNG4-SPP cells. Log phase promastigotes of WT, SPP+/-, WT+ SPP-GFP and SPP+/- +pXNG4-SPP (three clones) were processed for flow cytometry to measure their GFP fluorescence.
